# Environmental adaptations in metagenomes revealed by deep learning

**DOI:** 10.1186/s12915-025-02361-1

**Published:** 2025-08-11

**Authors:** Johanna C. Winder, Simon Poulton, Taoyang Wu, Thomas Mock, Cock van Oosterhout

**Affiliations:** 1 School of Environmental Sciences, University of East Anglia, Norwich Research Park, Norwich, NR4 7TJ UK; 2 School of Biological Sciences, University of East Anglia, Norwich Research Park, Norwich, NR4 7TJ UK; 3 BioEcoSS Ltd, 1 Granary Steps, Bridgnorth, Shropshire WV16 4BL, UK; 4 School of Computing Sciences, University of East Anglia, Norwich Research Park, Norwich, NR4 7TJ UK

**Keywords:** Deep learning, Transfer learning, Artificial neural networks, Metagenomics, Domain of unknown function 3494, Ice-binding proteins

## Abstract

**Background:**

Deep learning has emerged as a powerful tool in the analysis of biological data, including the analysis of large metagenome data. However, its application remains limited due to high computational costs, model complexity, and difficulty extracting biological insights from these artificial neural networks (ANNs). In this study, we applied a transfer learning approach using the ESM-2 protein structure prediction model and our own smaller ANN to classify proteins containing the domain of unknown function 3494 (DUF3494) by their source environments. DUF3494 is found in a diverse group of putative ice-binding and substrate-binding proteins across a range of environments in prokaryotic and eukaryotic microorganisms. They present a compelling test case for exploring the balance between prediction accuracy and interpretability in sequence classification.

**Results:**

Our ANN analysed 50,669 DUF3494 sequences from publicly available metagenomes, and successfully classified a large proportion of sequences by source environment (polar marine, glacier ice, frozen sediment, rock, subsurface). We identified environment-specific features that appear to drive classification. Our best-performing ANN was able to classify between 75.9 and 97.8% of sequences correctly. To enhance biological interpretability of these predictions, we compared this model with a genetic algorithm (GA), which, although it had lower predictive ability, provided transparent classification rules and predictors. Further in silico mutagenesis of key residues uncovered a vertically aligned column of amino acids on the b-face of the protein which was important for environmental differentiation, suggesting that both methods captured distinct evolutionary and ecological aspects of the sequences. Feature importance analysis identified that steric and electronic properties of the protein were associated with predictive ability.

**Conclusions:**

Our findings highlight the utility of deep learning for classification of diverse biological sequences and provide a framework for combining methods to improve model interpretability and ecological insights.

**Supplementary Information:**

The online version contains supplementary material available at 10.1186/s12915-025-02361-1.

## Background

Deep learning is becoming a powerful technology for complex modelling tasks in biology, which is being applied in studies on phylogenetics [1], biogeography [2] and protein structure predictions [3]. Input data for these models ranges from numeric to visual to language (text mining) and DNA/amino acid sequences. However, currently, much of the research using deep learning approaches with biological sequence data is restricted to high-level foundational tasks [4–6]. The potential to apply the high-level predictive capacity of deep learning to more specific downstream questions via transfer learning often remains restricted to simpler numeric categorical [7] and image-based datasets [8], especially outside of human biological contexts. Additionally, despite its often high predictive power, model outputs are difficult to interpret biologically, especially when little functional data is available. 

Recent sequence-based deep learning models operate on a scale of millions of diverse training sequences [9] or entire genomes [10]. Building and training models for these fundamental biological approaches is complex and has significant computational costs [11–13], limiting accessibility for smaller-scale applications. An alternative approach is to utilise transfer learning to adapt complex pretrained models to more specific tasks [14]. Transfer learning is the process by which a model developed for a general task is reused as the starting point for a model on a second, more specific task. For example, transfer learning of complex models pretrained on vast protein sequence datasets can repurpose the broad structural information learned by these large models to smaller scale tasks such as predicting specific protein thermostability [15]. These models also have the benefit of providing an alignment-free encoding method allowing comparisons of proteins with limited homology while avoiding the introduction of alignment-associated biases [16]. However, while transfer learning enables deep learning models to be repurposed for specific biological tasks, the inherent complexity and opacity of these models remain a significant challenge.

A major challenge in deep learning is the interpretability of the models and therefore their ability to provide biological insights [17]. Explaining the predictions made by deep learning models is known as explanatory AI (xAI). Complex deep learning models can be made more transparent by creating simpler, interpretable models, such as linear regressions to explain the data. Neural additive models aim to create an inherently interpretable combination of smaller neural networks via linear addition [18]. Glass box approaches such as ExplaiNN [19] build on this method for convolutional neural networks (CNNs). These CNNs encode DNA sequences (i.e. the nucleotide bases, A, C, T and G) by representing each nucleotide numerically as a vector such as [1,0,0,0] for A, [0,1,0,0] for C, (i.e. one-hot encoding). They use these vectors as input and return explainable models that predict genomic features. In contrast to the opaque inner workings of black-box models, such glass box models make the decision-making process more transparent. Alternatively, more traditional approaches measure how small, sequential changes in the input data alter the output. For sequence data, this can be done as in silico mutagenesis, and although effective [20], depending on sequence length and model complexity, it can also be time-consuming to mutate every residue in a sequence and predict outcomes [21].

Genetic algorithms (GAs) provide a transparent analysis on complex sequence data by finding predictive rules composed of logical expressions that can categorise the data. The stochastic optimisation algorithms of GAs contain all possible solutions in their search spaces, which means that they can effectively identify complex patterns to distinguish groups in data [22]. As such, GAs are complementary to ANNs and could help researchers interpret the decision-making process and features used in the data by ANNs. The GA used by Urban et al. (2024) and Smallbone et al. (2016) uses a form of discriminant function analysis to identify genetic variants responsible for phenotypic variation such as owl parrot plumage colour [23] or *Monogenea* infection resistance [24]. It is widely applicable as it can be used on any kind of categorical aligned sequence data. The advantage of a GA is that predictive rules are fully interpretable and can be understood in terms of logical expressions. As such, it is a powerful complementary tool to deep learning models by providing transparency.

Proteins containing the domain of unknown function 3494 (DUF3494) are a taxonomically diverse, environmentally widespread group that provide a test case for exploring the balance between prediction accuracy and interpretability [25]. DUF3494 is found in ice-binding proteins and substrate-binding proteins in microbial taxa across all domains of life, including organisms such as polar and temperate eukaryotic algae [26–28], bdelloid rotifers [29] and epiphytic bacteria [30]. The domain consists of a discontinuous left-handed β-solenoid braced by an alpha helix, and it has frequently been passed between taxa via horizontal gene transfer [31]. Proteins containing the DUF3494 have antifreeze activity in the form of thermal hysteresis (freezing point depression) and ice-recrystallisation inhibition [25]. These properties may confer freezing tolerance by preventing cellular damage and allowing the creation of habitable liquid environments for survival in subzero temperatures. It has therefore been the subject of extensive in vitro characterisation [26, 32], providing a solid biochemical foundation for interpreting model outputs. The structural basis of variable antifreeze activity in different DUF3494-containing proteins is an active area of research [33]. However, there are certain accepted paradigms, such as that features of importance include: an ice-binding site on the b-face of the β-solenoid containing hydrophobic amino acids with small side chains, a secondary column on the b-face containing hydrophilic residues possibly for hydrophobic interactions, stabilising interactions between the a-face and the alpha helix [34]. There may also be a direct role of the c-face in ice-binding [35]. The domain is found in protein families PF11999 and PF20597 which have highly similar structures but poor sequence homology [28]. PF11999 is found in most ice-containing and many ice-free environments (e.g. caves, lake sediment, hydrothermal vents) [36, 37]. Proteins in this family show some signals of environmental adaptation such as taxon-specific domain architectures, and large amounts of structural and sequence diversity even within a single ice-containing environment [38]. Large amounts of variation within and between environments create a complex multiclassification task which is well suited to a deep learning approach. Examining sequences containing this domain across a range of environments may reveal evolutionary adaptations that drive the high level of variability observed in this domain.

Here, we used a transfer learning approach to create an artificial neural network (ANN) that can classify DUF3494 sequences by their source environment. We collected 50,669 DUF3494 sequences from publicly available metagenomes as input for our feedforward ANN consisting of sequential dense layers. After testing different model architectures and hyperparameter optimisation, we assessed model performance. To shed light on the black box of the model, we examined characteristics of DUF3494 sequences, identifying putative ice-binding sites, conserved regions and overall patterns in sequence diversity. We utilised a genetic algorithm (GA) [23, 24] as an interpretable supplement to the ANN. We then performed targeted in silico mutagenesis based on results from the GA and classical sequence analysis to identify residues/structural features of importance for classifying DUF3494. Finally, we examined feature importance and correlated important features with physicochemical properties of the protein sequences. Our results provide an accessible test-case for using deep learning methods for the classification of complex biological sequences and shed light on environment-specific features of DUF3494.

## Methods

The aim of this study was to create an artificial neural network (ANN) that can classify DUF3494 sequences by their source environment, and to identify environment-specific sequence features that may inform on protein function and adaptation. We curated DUF3494 sequences from metagenomes and used a transfer learning approach to build a smaller feedforward artificial neural network (ANN). To interpret the model, we employed a genetic algorithm (GA) to extract interpretable rules, performed targeted in silico mutagenesis to test the impact of specific residues on model predictions, and investigated feature importance of the ANN. This study was conducted in silico using publicly available metagenomic datasets spanning a range of environments.

### DUF3494 sequence curation

DUF3494 sequences are involved in surface adhesion and ice-binding. To collect DUF3494 sequences from diverse substrates, a literature search was performed to collect environmental and biological surface-associated metagenomes. To obtain these surface-associated metagenomes, a Google Scholar search was performed. Search terms used can be found in Additional file 1: Table 1. Papers were filtered for publication post-2014, and the resulting 183 publications were manually scanned for correct data type (metagenomic), public availability of metagenomic sequences. Only Illumina sequences were selected to streamline computational processing. In total, data from 224 NCBI BioProjects were selected (Additional file 1: Table 2), comprising 6972 sequence read archive accessions.

In addition, 71 sea ice, seawater and sediment trap samples from the MOSAiC expedition in the central Arctic Ocean were analysed [39]. Sequence preprocessing up to producing gene models for the MOSAiC samples was performed by JGI, with any differences outlined below.

### Sequence preprocessing (Fig. 1)

Metagenomic sequences were downloaded from NCBI’s sequence read archive (SRA) using prefetch in combination with fasterq-dump (https://github.com/ncbi/sra-tools). Sequences were trimmed for adapters and filtered for quality using BBDuk from BBMap/38.86 (https://sourceforge.net/projects/bbmap/). Sequences were then assembled into contigs using metaSPAdes/3.15.5 [40]. In order to apply the appropriate gene modelling software, contigs were taxonomically classified as either “prokaryotic” “eukaryotic” or “unclassified” using kraken2 [41], and sorted accordingly. Gene prediction was performed using Prodigal/2.6.3 (prokaryotic and unclassified) (https://github.com/hyattpd/Prodigal) and MetaEuk v6 (eukaryotic) [42]. Modelled genes were then scanned against the PFam-A sequence profiles using hmmsearch from hmmer/3.3 (http://hmmer.org). The output was then filtered for the DUF3494-containing PF11999 (ice-binding like) and PF20597 (ice-binding like adhesive), and only genes containing these sequences were isolated. Genes were then filtered down to the hmmsearch hit region for the respective PFs (envelope start and end position) which should contain the DUF3494 region.

Sequences were filtered by length and *e*-value; all DUF3494 hit regions < 150 bp and any sequences with a hmmer domain e-value of > 1 × 10^−10^ were removed. Any genes which contained more than one DUF3494 domain were renamed to add unique identifiers so that each individual domain had a unique label.

Due to the lack of sequence homology between PF11999 and PF20597 [28] and more extensive functional information availability, only PF11999 was analysed here.

Taxonomic assignment of genes annotated as PF11999 was performed using MMSeqs2 [43] against the RefSeq90 [44] and MarFerret [45] databases. 

**Fig. 1:**
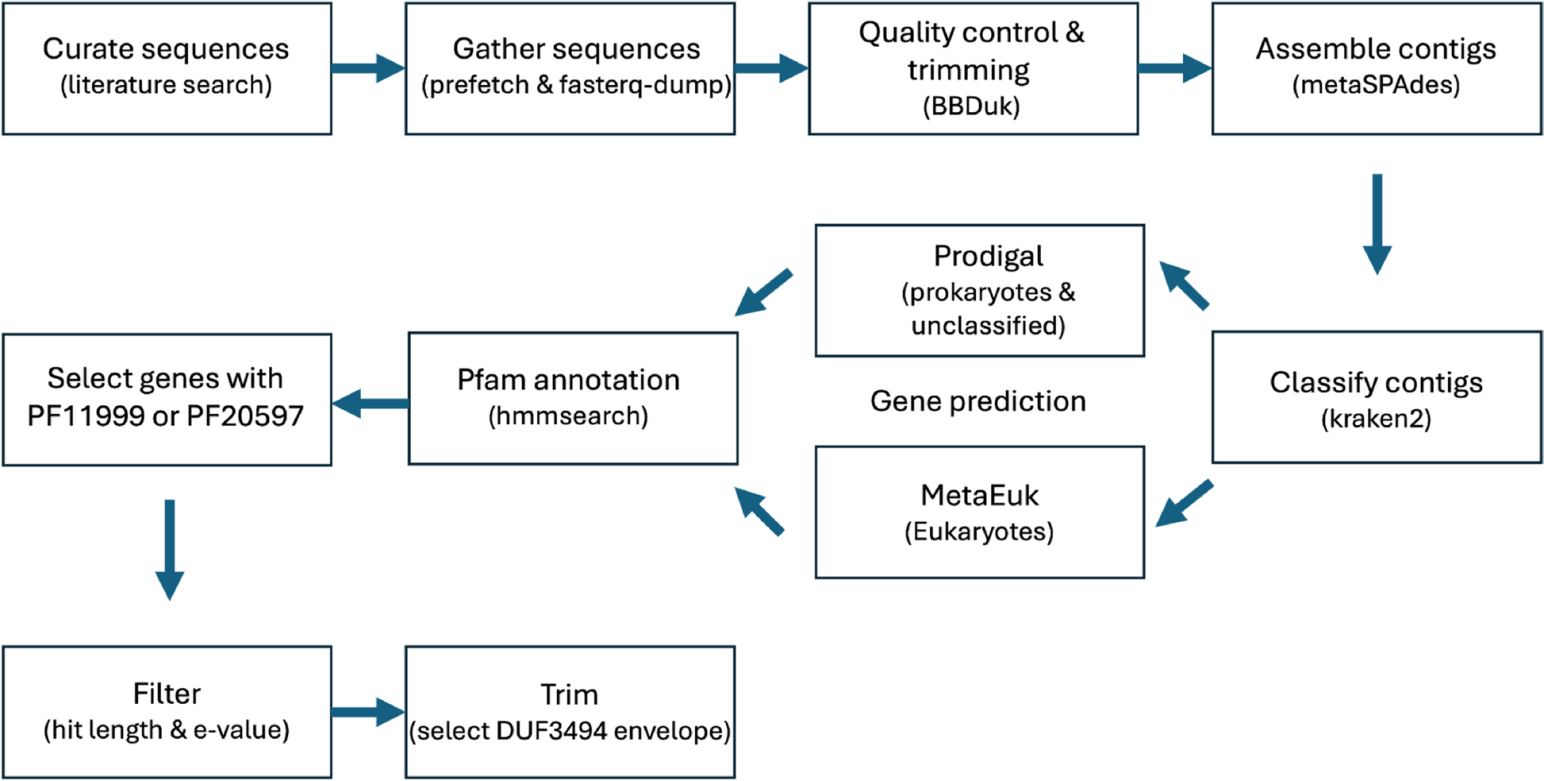
Bioinformatics workflow for curation and acquisition of DUF3494 sequences. Code for each step of this workflow is available at 10.5281/zenodo.16266612. Sequencing datasets were identified using an exhaustive literature search comprising 156 journal articles, and they were manually filtered for correct data types. Sequences were gathered using prefetch and fasterq-dump from the SRA toolkit. Sequences were cleaned and trimmed using BBDuk. Short read sequences were assembled into longer contigs using metaSPAdes. Each contig was taxonomically classified as either eukaryotic, prokaryotic (bacteria and archaea) or unclassified. Eukaryotic genes were identified in eukaryotic contigs using MetaEuk, while prokaryotic and unclassified genes were identified in their respective contigs using Prodigal. Gene models were then translated to amino acids and annotated with their protein families using hmmsearch. Genes containing DUF3494 were selected and filtered for hit length > 150AAs and *e*-value < 1 × 10^−10^. Finally, these hit regions (envelopes) were selected from the larger protein sequence for input into downstream applications. Only PF11999 was used for downstream analyses

### Data exploration of PF11999 DUF3494s

An alignment of all DUF3494 sequences was generated as input for the genetic algorithm, benchmarking the ANN, and for use in interpreting outputs of the NN. Due to the large number of sequences (~ 50,000) and their diversity, traditional methods (ClustalO, MAFFT) produce inflated alignments that are many times the size of the actual gene [46]. These methods are also computationally too intensive for large metagenome datasets like ours. We therefore used a method outlined in [47] for producing large alignments, using MAGUS and ensembles of hidden Markov models (MAGUS + eHMMs). Briefly, a random subset of 1000 sequences was selected to form a backbone alignment using MAGUS [48]. A tree was generated from this backbone alignment using FastTree [49]. UPP (Ultra-large alignments using Phylogeny-aware Profiles) [46] was then used to break the backbone into HMMs to align the remaining sequences against. The resulting alignment was then filtered to remove gaps. Initially, positions in the alignment which were > 50% gaps were removed from the alignment. Following this, sequences which were composed of > 30% gaps were removed from the dataset.

Positions of this filtered alignment were then mapped back onto the 3D structure of the DUF3494 domain by selecting 50 sequences with the least gappy alignments, running structural predictions with ESMFold and manually assigning alignment positions a structural label (e.g. a-face, b-face, c-face, between faces, alpha helix).

The most conserved regions across the alignment were identified by plotting the rolling average of Shannon diversity (Additional file 2) across three positions. A given position was considered conserved if it (a) had a rolling average Shannon diversity of < 1.50 and (b) the following position did not have a Shannon diversity of more than 0.1 lower than it. This way, we ensured that a position with average Shannon diversity below the cutoff threshold was not just the result of a single position in the rolling average window.

### ESM-2 encoding

Encoding is a fundamental step in data preparation for machine learning models, as it defines how the model processes categorical information (such as language), thereby also guiding the model’s capacity for learning new features of the data. For example, integer encoding of amino acids (AAs; letters, unprocessable by a model) as numbers (1–21) could bias the model to weight AA 21 more than AA 1 due to the higher number. In order to ensure the model was provided with sufficient information about the AA sequence, to allow it to learn other features about the sequence (such as information about secondary structure, tertiary structure, physicochemical properties), a transfer learning approach was used.

In transfer learning, a model that has been developed for a general task is reused as the starting point for a model on a second, more specific task. The output or intermediate layers of the initial model are repurposed for a more specific task with a potentially smaller training set. In this case, transfer learning was used to take the penultimate (33rd) layer of Meta Fundamental AI Research’s Evolutionary Scale Modelling 2 (ESM-2) [9, 50, 51], and the output of the 33rd layer was then used as input for our more specific classification task. Here, we refer to this as L33 encoding.

In the ESM-2 model, sequence inputs are passed through a deep Transformer encoder model which uses one-hot encodings of AAs, and learns additional positional embeddings through rotary position embedding [52], initially outputting a vector of log-probabilities for each position in the sequence [53]. These log-probabilities are then fed into a language model which is trained in an unsupervised manner. During this task, 15% of each training sequence is masked and the language model is tasked with predicting the missing residues [9, 53]. The model thereby learns internal representations of the input sequence, leading to a language representation of the sequence [53, 54].

The folding module of the model was trained on 325,000 experimentally determined protein structures and 12 million protein structures predicted by AlphaFold2 in Protein Data Bank (PDB) format [9]. In this folding module, residue-residue contacts are learned, with the output being a 3D structural prediction for the protein.

### Transfer learning based on ESM-2

We extracted the average of per-residue representations from the 33rd layer of ESM-2 (L33), which encodes information about the 3D structure of the protein, as well as information about the chemical properties, secondary structures and other features of the protein sequence. This layer contains a set of 1280 features for each amino acid in the sequence that capture learned features of the protein. We have averaged these into 1280 features for the whole protein sequence, as in [55] as our encoding. Embeddings from the penultimate 33rd layer are independent of protein length. In contrast, the final layer outputs a PDB file that is sequence-length dependent and less machine-readable [56]. Hence, using the average per-residue representations for the penultimate layer is a generally recommended approach for transfer learning [55]. L33 encoding was obtained using the fair-esm package in Python (https://github.com/facebookresearch/esm).

### Artificial neural network architecture (Fig. 2)

**Fig. 2:**
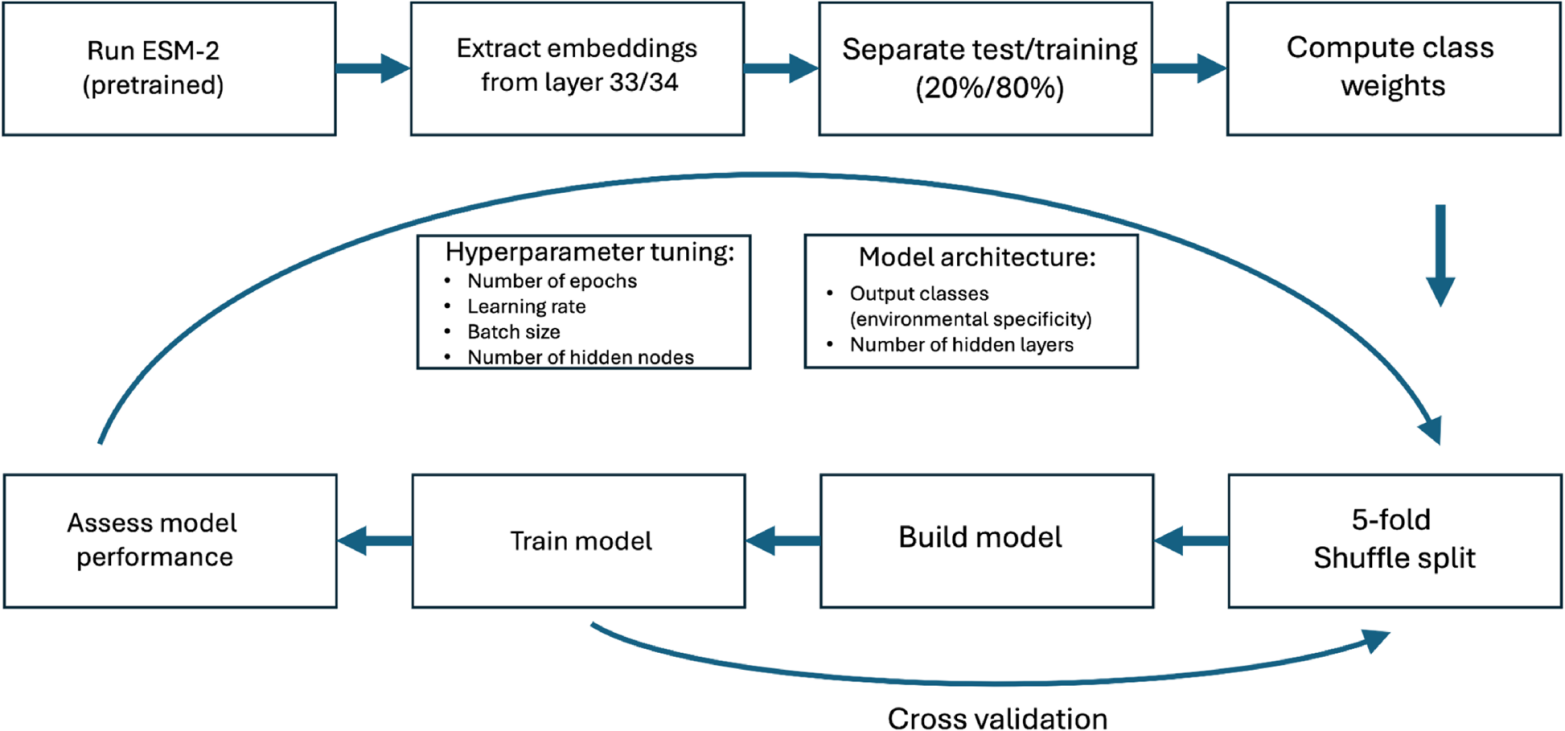
Building and training an artificial neural network (ANN) to classify PF11999 DUF3494 sequences by environment. We ran FAIR's Evolutionary Scale Model 2 and extracted the average per-residue model weights from the penultimate layer (L33) as the input for our own smaller ANN. Sequence embeddings were separated into test (20%) and training (80%) datasets, and the test data was withheld to assess model predictive capacity after training (see the “Methods” section). ANN performance was assessed on the training set and the process was repeated for different hyperparameter values and model architectures, with training/validation accuracy and loss recorded each time. Code is available at 10.5281/zenodo.16266612

L33 encoding was used as the starting point for our specialised artificial neural network (ANN) (Fig. 2). While the L33 model had the task of predicting the 3D structure of a protein given an AA sequence, our smaller ANN had the task of classifying sequences into their source environments. We investigated a few model architectures with different levels of output specificity (the number of target environment types for classification) and training methods.

The ANN we have developed is a sequential model with two hidden dense layers containing 800 and 400 nodes respectively. ANNs contain input layers, hidden layers and output layers. The input layer is the L33 encoded AAs; that is, the 1280 feature vectors obtained from the penultimate 33rd layer in the ESM model, while the output layer is the predicted environment type. The hidden layers are the layers between the input and output layers. For each node in these dense hidden layers, every input feature for this layer is multiplied by a different weight, resulting in a weighted sum of all input features. This weighted sum has a bias term added and is further passed through an activation function known as ReLU [57]. This function maps a negative number to 0 and a positive number to itself; therefore, it introduces nonlinearity that allows the model to learn complex patterns in the data.

In the output layer, there is a node designed for each of the output classes. The outputs of the last hidden layer are passed through the output activation function, which normalises these raw scores into probabilities, generating a probability distribution across all output classes for each input sequence. The node with the highest probability then becomes the predicted class. For binary classification, the output activation function would be logistic, as this has the appropriate probability distribution. For multiclassification (i.e. > 2 classes), the output activation function is softmax (multinomial distribution) for the same reason.

ANNs learn by adjusting the weights in the hidden layer to optimise performance. Internally, performance is measured using a loss function, which represents the difference between the model output and the target output (i.e. the labels in a training dataset). A categorical crossentropy loss function was used for multiclass classification (for further explanation of this function see Additional file 2). To minimise loss, we used the Adam (adaptive moment estimation) optimiser [58]. Adam uses the second derivative of the loss function to identify when its rate of change slows, and slows the search progression (learning rate, see below) accordingly to find a minimum. We tested three models with various hyperparameter combinations (Additional file 1: Table 3) to select environment specificity and tune hyperparameters.

### Testing environment specificity levels

We tested four levels of environment specificity with the goal of finding the model which was the most accurate to the highest level of environment specificity (Table 1).

**Table 1:** Sample distributions for different environment types and their labels under different environmental splits. Samples were grouped into five environment labels based on scientific relevance, environment abundance and model misclassification patterns. Samples which did not fit into these groups and which had < 1/100 abundance of the largest grouping were removed from Model 3. The large class imbalance(especially between polar marine and subsurface) was accounted for in the model using class weights to penalise prediction of more abundant environments

Environment	# of samples	Model 1 (11 labels)	Model 2 (8 labels)	Model 3 (5 labels)
Sea ice	23,277	Sea ice	Polar marine	Polar marine
Melted sea ice	4171			
Arctic seawater	2623	Arctic seawater		
Arctic ocean sediment	2578	Marine sediment		
Permafrost soil	1212	Frozen sediment	Frozen sediment	Frozen sediment
Cryoconite	5989			
Glacier sediment	362			
Glacier ice	2427	Glacier	Glacier	Glacier
Glacier snow	2399			
Glacier surface	161			
Polar endolith	7787	Rock	Rock	Rock
Rock	32			
Rock varnish	30			
Deep subsurface	392	Subsurface	Subsurface	Subsurface
Cave sediment	61			
Cave groundwater	110		Cave groundwater	NA
Aquatic sediment rhizosphere	106	Aquatic plant	Non-frozen sediment	
Aquatic phyllosphere	25		NA	
Aquatic leaf	24			
Aquatic root	53			
Soil rhizosphere	102	Terrestrial plant	Non-frozen sediment	NA
Phyllosphere	1		NA	
Marine biofilm	36	Biofilm	Biofilm	
Microbial mat	29			
Glacier fed lake water	57	Glacial lake	NA	
Subglacial lake	4			
Hydrothermal vent	1	NA	Non-frozen sediment	
Human oral	1	NA	NA	

### Hyperparameter testing for artificial neural network (ANN)

We tested hyperparameter combinations for: number of training epochs, batch size and learning rate (for further explanation, see Additional file 2). Additionally, we tested two different model architectures: two layers with 800/400 hidden nodes and one layer with 800 hidden nodes. For all hyperparameter testing, the model was trained using TensorFlow [59].

Model performance was assessed using average accuracy and average training loss. Models with the lowest average loss and highest accuracy combinations were then assessed with a confusion matrix. The confusion matrix identified the true positive and true negative rates, differentiating specifically which outputs were misclassified into which classes. The high performing model, which was the most consistently accurate across classes, was then considered the overall “best performing” model. The goal was to identify which model(s) were not only overall the best performing but which successfully characterised sequences from every environment correctly more often than misclassifying them. Models which misclassified any environment more often than they classified it correctly would be discarded on the basis of the confusion matrix.

### Running the artificial neural network (ANN)

The model was trained using TensorFlow [59]. Test and training datasets were separated into mutually exclusive groups using StratifiedShuffleSplit from scikit-learn, with a single 80%/20% split. The training data was then used to build the model, while the test data was held aside. As our aim was to gain biological insights into an existing dataset, rather than test predictive power, we followed the standard approach of selecting an unseen subset of our dataset as the test dataset, rather than curating and processing a completely separate dataset.

Stratified sampling was used to minimise the effect of class imbalances. Additionally, to account for class imbalance, we applied class weighting during model training. A penalty proportional to the number of sequences in each environment was assigned, ensuring that lower-abundance classes received greater emphasis. This weighting mechanism helps mitigate biases introduced by imbalanced training data and improves classification performance across all environmental categories. Class weights were computed using compute_class_weight from scikit-learn. fivefold cross validation was performed using KFold from scikit-learn. The model was a sequential model containing only fully connected (dense) layers: the input layer, two hidden layers and the output layer. The input layer was the average per-residue L33 encoding for the DUF3494 AA sequence. The first hidden layer was a dense layer with 800 neurons (hidden nodes) and used the ReLU activation function. The second hidden layer was a dense layer containing 400 hidden nodes, also using the ReLU activation function. A dropout rate of 50% was applied to each hidden layer during training—that is, at each training step, half of the neurons in each hidden layer are randomly selected and their outputs set to zero. The output layer contained five units (the number of output classes) and used the softmax activation function. The model used categorical crossentropy as its loss function, and the Adam optimiser (tensorflow-keras) with 1 × 10^−4^ learning rate was used. The model was trained for 200 epochs with a batch size of 512.

### Benchmarking the ANN

Our ANN was benchmarked against hidden Markov model (HMM) approaches and logistic regression approaches. For the HMM approach, we randomly sampled 80% of sequences from each environment. These sequences were aligned using MAGUS + eHMMs as described above to produce HMMs. This set of HMMs was concatenated, and the remaining 20% of sequences were then searched against it using hmmsearch from hmmer/3.3 (http://hmmer.org). The best hit was determined by the highest bit score for the full sequence. We then compared the predicted environment (best hit) to the actual environment to compare performance to our ANN.

We compared ANN performance against 2 logistic regression models: one using positional identity as input, and one using L33 encodings as input. For the positional model, the MAGUS + eHMM alignment generated in Sect. 3 was used as input. Each position in the alignment was one-hot encoded according to a dictionary of all possible amino acids + a gap character (21 possible values). This resulted in 3885 (21 × 185) input features. Inputs were split into test and training datasets in the same manner as for the ANN, described above. A multiclass (5) logistic regression model was built in Python using LogisticRegression from scikit-learn. The model used the saga solver with L2 regularisation and 6000 iterations and included the same class weighting as the ANN. The same modelling approach was used for logistic regression of L33 encodings, but with 2000 iterations. The models were assessed for accuracy, F1, precision and recall on the test dataset.

For proof-of-concept regarding building a version of our ANN to classify unalignable sequences, we also generated a model which included both known DUF3494-containing Pfams: PF11999 and PF20597. The proteins were L33 encoded; the model was trained for 500 epochs with a batch size of 512. This version of the model included frozen sediment, natural biofilm, rock and subsurface environments. We also generated sample protein structure predictions using ESMFold and sample multiple sequence alignments using ClustalOmega to demonstrate the limited sequence homology between, compared to within, these protein families.

We assessed phylogenetic signal in the data using a multinomial logistic regression model of environment type ~ phylum. Only sequences which had a phylum-level taxonomic classification were included in this model. The association between environment type and phylum were assessed versus a null model using McFadden’s pseudo-*R*^2^. The nnet package in *R* [60] was used for multinomial regression, and the performance [61] package was used to calculate *R*^2^. We produced a phylogenetic tree of DUF3494 sequences using FastTree [49], and visualised it using ggtree [62].

### Feature importance

Feature importance was used to identify features which might be important in differentiating DUF3494 sequences from different environments. Shapley additive explanations (SHAP) were used to identify which features were overall the most important for model predictive capacity. This method builds off of Shapley values: a method from coalitional game theory for assigning payouts to individual players based on their contribution to the overall payout [63]. In the context of an ANN, they represent a weighted average marginal contribution of a feature to the overall prediction. SHAP is an extension of these Shapley values which allows for better global interpretability [64] by translating Shapley values into an additive feature attribution model. SHAP was performed with the SHAP package in Python (https://github.com/shap). The features with the highest magnitude of importance were selected for feature interpretation.

The effects of phylogenetic structure on model classification ability were characterised using a binomial logistic regression model of ANN classification correctness (correct/incorrect) ~ phylum. Only sequences which had a phylum-level taxonomic classification were included in this model. The association between classification correctness and phylum was assessed versus a null model using McFadden’s *R*^2^.

### Running the genetic algorithm (GA)

The custom genetic algorithm is a generalised form of Discriminant Function Analysis (DFA). It takes a matrix where the rows are sequence identifiers and columns are predictor variables. These predictor variables can be amino acids in aligned sequences. An additional column represents the target variable, in this case, an environment label. The algorithm starts by generating a set of random expressions based on the predictor variables, such as (position 50 = A). This is then evaluated against each sequence as true or false. The algorithm can also generate compound expressions that are linked with logical operators; for example, ((position 50 = A) AND (position 73 < > T) OR (position 102 = I)). Each of the random expressions is then scored against the target expression (e.g. *R* = True) using a penalised phi-coefficient. The lowest scoring rules are discarded—the remainder being preserved as the “breeding” population. The algorithm then loops through a user-defined number of “generations”, using the breeding population from which to mutate or recombine new predictive expressions, plus a number of entirely new random expressions. These are evaluated as described above, and the populate of expressions “evolves” to give the best set of predictive expressions. The algorithm is written in Transect-SQL and implemented in Microsoft SQL Server version 16.0.1121.4. The GA was run for 2500 generations. Two iterations of the GA were run. For the first batch of analyses, the phi penalty was relaxed to allow expressions to contain up to 20 parameters, and for the second, it was restricted to just two parameters.

### Model interpretation

To interpret model outputs and shed light on the biological drivers of its predictive capacity, we correlated important features with physicochemical parameters and used an in silico mutational approach. Firstly, we correlated the three most important features identified by the feature importance analysis above with the 50 physicochemical parameters which comprise vectors of steric and electronic properties (VHSE8) [65] to assess the biological significance of these features. This correlation analysis was performed on a stratified 100-sequence subsample (equal proportions for each environment) of the DUF3494 dataset. The value of each physicochemical parameter for a given amino acid was taken from the Amino Acid Index Dataset (genome.jp) and averaged for each sequence. The percentage explained variation (R2) was calculated using a Linear Regression analysis for each feature versus average physicochemical property of the entire amino acid, as well as for each of the faces of the protein.

We also constructed in silico mutant sequences, randomly replacing amino acids based on residues of importance identified with the GA (Additional File 2). The full-length in silico mutants were then run through the L33 encoding process as described above. The ANN was then used to predict the environment of each of these new sets of sequences, as well as their original counterparts. The accuracy and misclassification pattern of these predictions were then used to identify whether a given region of the protein had a larger impact on the predictive ability of the model.

## Results

### Artificial neural network (ANN) output

**Fig. 3:**
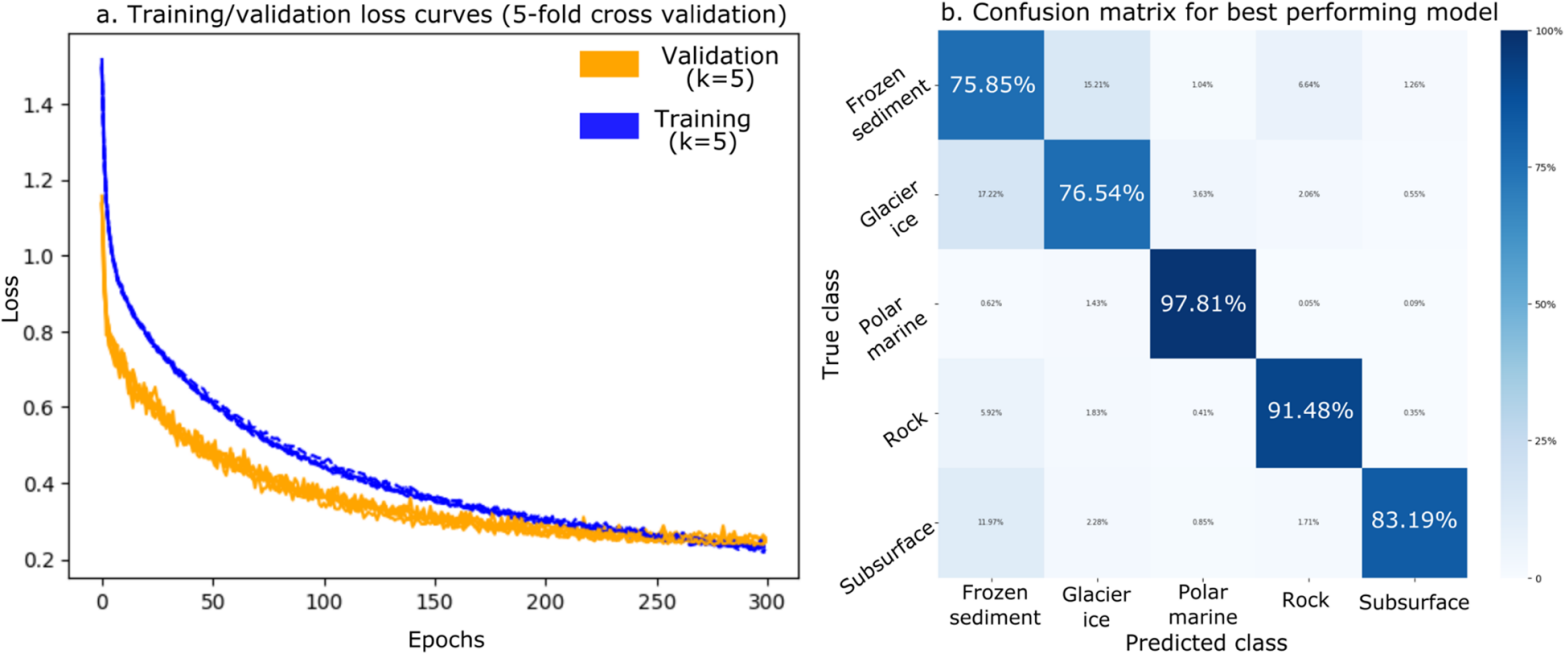
An artificial neural network (ANN) is able to classify PF11999 DUF3494 sequences by environment with 92.0% accuracy and minimal overfitting. **a** Training (blue) and validation (orange) loss curves for the fivefold cross validation training of the best performing ANN with 2 hidden layers (800 and 400 hidden nodes respectively), batch size of 512 and learning rate of 1 × 10^−4^, trained for 300 epochs a dropout rate of 50% applied to each hidden layer during training. The *X*-axis is the number of training epochs (max 200) and the *Y*-axis is the loss value. Lines cross around 100 epochs with training loss continuing to decrease at a faster rate than validation loss. Model training is stopped when validation loss is no longer decreasing. **b** Confusion matrix for the best performing model shows that frozen sediment, glacier ice and subsurface sequences may share features that make them difficult to distinguish. Predicted class is on the *X*-axis and true class is on the *Y*-axis. Squares are coloured according to the percentage of that class’s true sequences which were predicted as the corresponding *X*-axis class. Darker blue corresponds to a higher proportion of sequences. Frozen sediment sequences were correctly classified 75.9% of the time, and were most often misclassified as glacier ice. Glacier ice sequences were correctly classified 76.54% of the time and were most often misclassified as frozen sediment. Polar marine sequences were correctly classified 97.81% of the time and were rarely misclassified. Rock sequences were correctly classified 91.5% of the time and were most often misclassified as frozen sediment. Subsurface sequences were correctly classified 83.2% of the time and were most often misclassified as frozen sediment

We trained our ANN on 50,669 PF11999 DUF3494 sequences spanning five distinct environmental contexts: frozen sediment, rock, polar marine, subsurface and glacier ice. The best performing ANN had two hidden layers with 800 and 400 hidden nodes respectively. It contained five target environments: frozen sediment, rock, polar marine, subsurface and glacier ice. Batch size was 512 and learning rate 1 × 10^−4^. The model was trained for 300 epochs to minimise overfitting. Mean accuracy of this model for test sequences was 92.0% with mean validation loss of 0.248, identical to the mean training loss of 0.201 (Fig. 3a) indicating stable learning across sequence types. The average per-environment accuracy was 85.0% ± 9.6%. Classification accuracy varied between environments, with frozen sediment being least accurately classified (75.9%) and polar marine being the most accurately classified (97.85%) (Table 2; Fig. 3b). Notably, classification accuracy for subsurface sequences ranked middle among the five classes (83.19%) despite this class having the fewest data points.

**Table 2:** Precision, recall and F1 for best performing artificial neural network. Precision refers to the number of true positives divided by the number of sequences predicted as that class. Recall refers to the number of true positives divided by the number of elements actually in that class. F1 score refers to the harmonic mean of the precision and recall

	Neural network		
Class	Precision	Recall	F1 score
0: Frozen sediment	0.80	0.75	0.77
1: Rock	0.92	0.92	0.92
2: Subsurface	0.78	0.82	0.80
3: Polar marine	0.99	0.98	0.99
4: Glacier ice	0.67	0.78	0.72

Misclassification patterns from ANN predictions imply that there may be shared feature(s) between sequences from frozen sediment, glacier ice and subsurface sequences that can make them difficult to distinguish from each other, but not rock and polar marine sequences (Fig. 3b). The most common misclassifications were glacier ice sequences that were being predicted as frozen sediment (17.22% of glacier ice sequences), frozen sediment sequences being predicted as glacier ice (15.21% of frozen sediment sequences), and subsurface sequences being predicted as frozen sediment (11.97% of subsurface sequences).

Class weighting and stratified sampling during training with k-fold cross validation were applied to account for imbalance in the number of samples in different environments. The inclusion of class weights improved classification accuracy for subsurface samples from 74.23% without class weights to 83.19% with class weights, but did not affect overall model accuracy.

We benchmarked our ANN against a number of other multiclassification approaches: a HMM-based approach, logistic regression (LR) based on positional identity (LR-PI), and LR based on L33 encoding (LR-L33) (Additional file 3). For our HMM-based approach, 80% of sequences per environment were used to generate HMMs, and accuracy was assessed based on which environment had the highest bit score for the remaining 20% of sequences. This approach had an average 49.7% ± 28.7% per environment accuracy. A multiclass LR-PI model with L2 regularisation performs slightly worse compared to our ANN in terms of average accuracy on an unseen test set (88% for LR-PI versus 92% for the ANN). The average per environment accuracy is also lower compared to our ANN (80.6% ± 11.6% for LR-PI versus 85.0% ± 9.6% for the ANN). Importantly, our ANN uses alignment-free encoding—specifically, embeddings from the ESM model—which do not require the sequences to be of equal length or to share sequence similarity. This enables the model to be applied broadly to variable and potentially divergent sequences. In contrast, any method that uses positional identity directly as input, such as the LR-PI model or our genetic algorithm approach, inherently requires sequences to be the same length. This can be achieved through trimming, padding, or alignment (sequence or structure), which has several downsides including computational feasibility (see Additional file 3 for further details and comparisons). To conceptually demonstrate this, we include a version of the ANN which includes PF20597, which shares limited sequence homology with PF11999 (Additional file 3).

### Features of DUF3494 sequences

To investigate whether PF11999 DUF3494 sequences bear environment-specific signatures that reflect adaptation, we examined biologically relevant residues across the protein’s structural faces (Table 3), assessed levels of sequence conservation and used ordination analyses to explore natural clustering of sequences by environment.

**Table 3:** Conserved regions of DUF3494s fell on all three faces of the β-solenoid but not the α-helix. Positions in the DUF3494 alignment which correspond to regions of the β-solenoid. The β-solenoid contains rows of β-sheets which are aligned horizontally and wrap around to form the solenoid. β-sheet position refers to which row number down the face of the solenoid the position is found on, while protein face refers to which face (a, b, c or between) of the protein the residue is found on

Position in DUF3494 alignment	β-sheet position	Protein face
5–10	2nd from top	b
17–20	2nd from top	a
26–30	Top	b
71–73	Bottom	b
76–78	Bottom	c
80–83	Bottom	a
94–98	2nd from bottom	c
105–109	2nd from bottom	a
124–127	2nd/3rd from bottom	c/a
132–135	3rd from bottom	a
142–145	4th from bottom	b
151–154	4th/5th from bottom	c/a
169–173	5th/6th from bottom	c/a
176–180	6th from bottom	b

#### Overall diversity

**Fig. 4:**
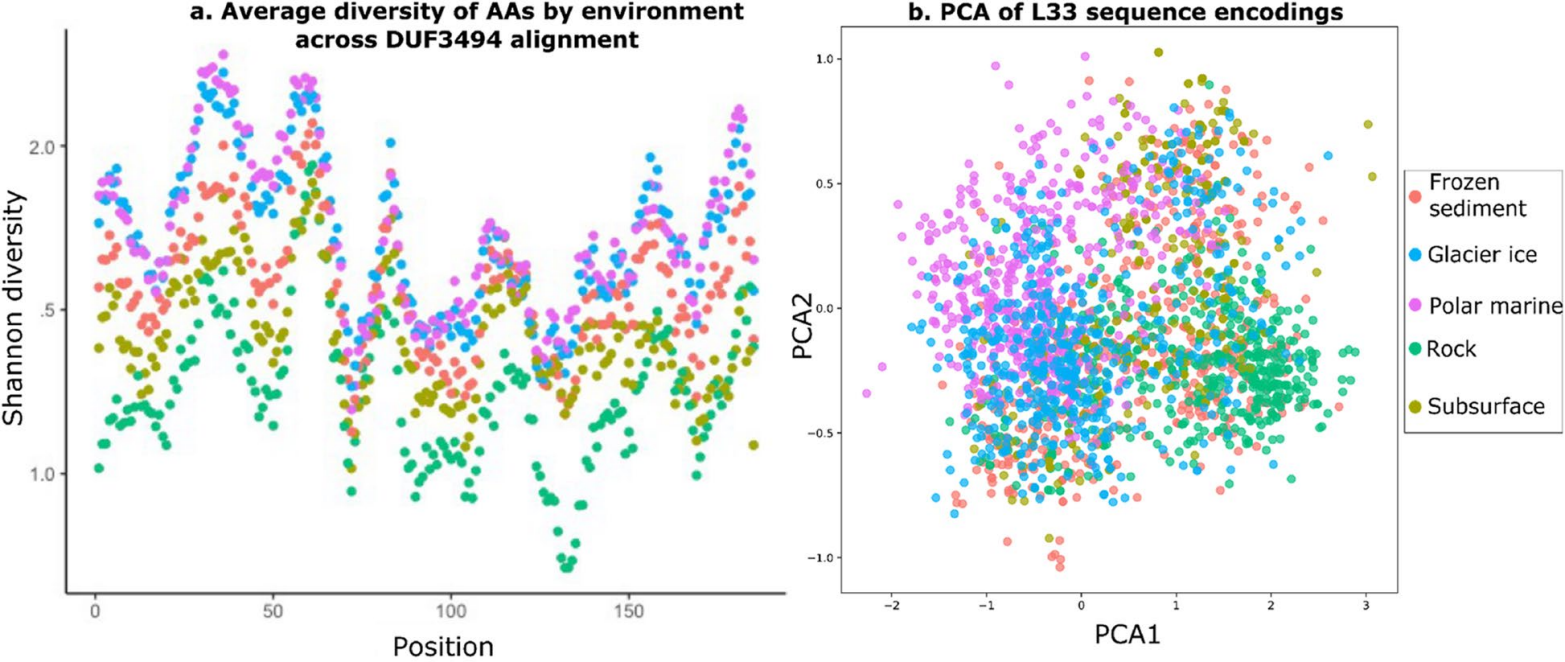
PF11999 DUF3494 sequences show different levels of sequence conservation across the length of the protein and overall between environments, but do not cluster distinctly by environment with a PCA. **a** Rolling average of Shannon diversity (Formula 1) of AAs by environment across a filtered alignment of DUF3494 sequences. Rock has the lowest diversity overall while glacier ice and polar marine have the highest. **b** Principal components analysis of L33 encodings of DUF3494 sequences shows complex overlapping clustering of sequences from different environments. Note that each environment has been subsampled down to ≤ 500 sequences per environment for visibility

Amino acid diversity of the domain varied significantly between environments, with the most conservation being observed in rock-associated environments. After adjustment for position, sequences from rock-associated environments had the lowest diversity, and polar marine and glacier ice having the highest (Fig. 4a) (ANCOVA; *F*_4, 919_ = 27.92; *p* < 0.001). When L33 encodings of sequences (vectors of 1280 features per sequence) were reduced via PCA with 3 components, sequences could not be clearly differentiated by environment (Fig. 4b, Additional file 4: Fig. 1).

#### Phylogenetic signal

Environmental patterns in DUF3494 sequences may reflect lineage-specific ecological adaptations. We examined the relationship between phylum-level taxonomy and environment type to determine whether taxonomy predicts the environmental distribution of DUF3494 proteins. Of the genes annotated with PF11999, 44.03% could be assigned to a phylum, an average of 40.4% ± 16.3% of sequences in each environment (Additional file 4: Fig. 2). 42.45% of classifiable sequences were found in the polar marine environment despite just 32.1% of polar marine sequences being classified to the phylum level, as a result of the large number of DUF3494 sequences found in this environment. The subsurface environment had the highest proportion of classifiable sequences (75.3%) while rock had the highest proportion of a single phylum (Actinomycetota; 42.2% of sequences). Using a multinomial logistic regression model with phylum as the sole predictor, and adjusting for class imbalance via class-weighted likelihood estimation, we found that the model explained 48% of the variation in environment type (McFadden’s *R*^2^ = 0.480) after unclassified sequences were removed. This strong association suggests that some classifying patterns in DUF3494 sequences may be a result of evolutionary constraints, leading to a phylogenetic signal.

#### Important residues

**Fig. 5:**
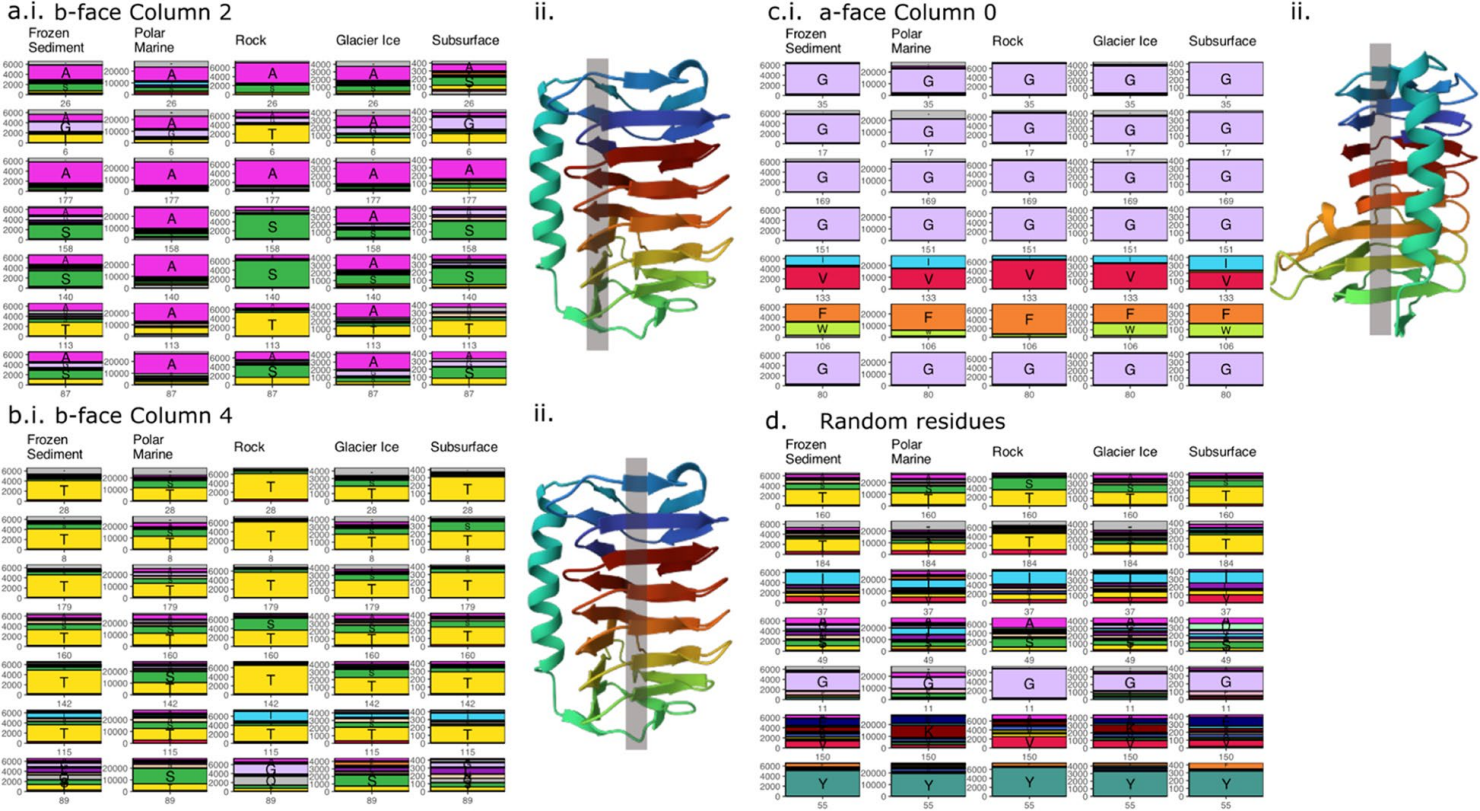
Some columns of interest show environment-specific trends while others are consistent between environments. **a.i**. Column 2 on the b-face shows higher frequency and consistency of alanine (A) in polar marine and glacier ice environments while frozen sediment, rock and subsurface environments contain higher proportions of other amino acids (AAs), especially serine and threonine. The bottom residue in the column (residue 87) is the most diverse across all environments except polar marine. **a.ii.** Location of column 2 residues on the b-face of the protein. **b.i.** Column 4 on the b-face is dominated by threonine in all environments, but this is less consistent in polar marine and glacier ice environments. The bottom residue in the column (residue 89) is the most diverse across all environments except polar marine. **b.ii.** Location of column 4 residues on the b-face of the protein. **c.i.** Column 0 on the a-face is consistent across environments, containing glycine residues for four rows, followed by isoleucine/valine and phenylalanine/tryptophan, and a final glycine. **c.ii.** Location of column 0 residues on the c-face of the protein. **d** Randomly selected residues do not show the same environmental differences or consistency as the columns, supporting that the previous panels illustrate biologically meaningful properties with AA sequences that are adapted to specific environments

To identify potential signatures of environmental adaptation at the molecular level, we examined variation in specific amino acid positions across environments, focusing on 16 putative ice-binding sites that align vertically in “columns” along the b and c faces of the DUF3494 protein (Table 4; Additional file 3). We compared all columns on the b and c faces of the protein (Fig. 5) to identify putative ice-binding columns. As we were interested in positions where a small number of AAs (either T/S or A/G) were dominant, we calculated the Shannon diversity index (H) of each position. We then selected the three columns with the highest proportion of their most abundant AA (Fig. 5e) and performed per-environment calculations on these columns.

**Table 4:** Identifying columns of interest across the 3 faces of PF11999 DUF3494 proteins. Columns is a structural term used to refer to vertical lines of amino acids down the 3 faces of the protein. We partitioned the faces into their columns and calculated Shannon diversity and the proportion of the single most abundant AA in that column in order to identify columns that were putative ice-binding sites. Columns with the lowest Shannon diversity and highest proportion of their most abundant AA were selected for further analysis (highlighted in blue)

Shannon diversity	Proportion of most abundant AA	Protein face	Column #
2.41	0.27	b-face	col_0
1.83	0.37	b-face	col_1
1.88	0.47	b-face	col_2
1.99	0.27	b-face	col_3
1.94	0.42	b-face	col_4
2.35	0.23	b-face	col_5
2.38	0.26	b-face	col_6
2.33	0.19	c-face	col_0
2.49	0.25	c-face	col_1
2.12	0.27	c-face	col_2
2.54	0.20	c-face	col_3
1.36	0.63	a-face	col_0
2.51	0.18	a-face	col_1
2.39	0.16	a-face	col_2
2.72	0.16	a-face	col_3
2.41	0.23	a-face	col_4

Analysis of three selected columns revealed environment-specific patterns, with column 2 especially highlighting functionally relevant distinctions between all five environments which may reflect ice-binding ability. Examining the three columns of interest identified that these columns were often the least diverse in the rock environment (Fig. 5). Column 2 (b-face) was most frequently occupied by S and A, sometimes T (Fig. 5a). While rock sequences were less diverse, the AAs were inconsistent down the column. In contrast, in polar marine and glacier ice environments, the column was consistently dominated by hydrophobic AAs. Column 4 (b-face) was most frequently occupied by S and T (Fig. 5b). Rock, subsurface and frozen sediment sequences consistently contained hydrophobic residues T/S down the column, while polar marine and glacier ice environments also contained large proportions of other AAs. Column 0 (a-face) was generally less diverse and more consistent, with minimal environmental differences (Fig. 5c). Figure 5d shows randomly selected residues to illustrate that they do not show the same environmental differences as the AAs in Columns 2 and 4, specifically.

### Genetic algorithm

**Fig. 6:**
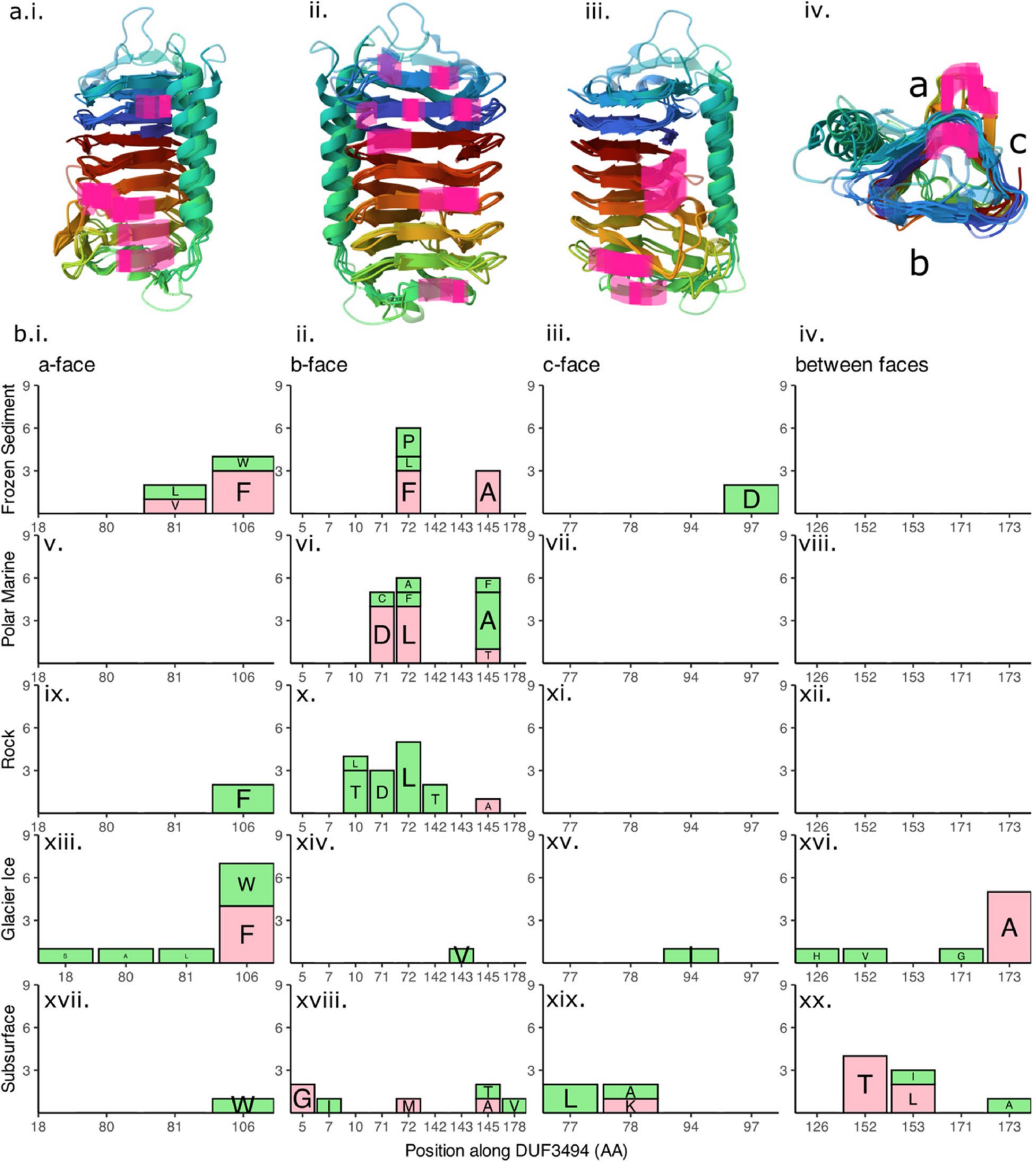
Positions and characteristics of AA rules for PF11999 DUF3494 sequences in different environments based on analyses with the Genetic Algorithm (GA). **a** Positions of amino acid rules highlighted on structural alignments of DUF3494 protein structures. **b** The presence and absence of certain AAs at different positions along DUF3494. Green and red refer to rule components which dictate that a specific amino acid is present (=) or absent (< >) at that position

To further identify environment-specific residues that may contribute to functional adaptation, we used a genetic algorithm (GA) to evolve predictive rules distinguishing sequences from different environments. The GA with a 20-parameter allowance was able to generate rules whose per-class accuracy was 72.3% ± 6.3%, which is less than the ANN (85.0% ± 9.6%). This predictive capacity was lowest for glacier ice (64.2% ± 2.4%) sequences and highest for polar marine sequences (78.7% ± 2.3%) (Additional file 1:Table 5). The most common positions of rules were positions 71 (29 rules) and 72 (22 rules) (Additional file 5). These positions correspond to the bottom loop of the predicted b-face of the β-solenoid (Fig. 6). The full rules are in Additional file 1:Table 5. 

When the GA was constrained to generate rules involving only up to two amino acids, predictive capacity was retained, revealing a consistent role for hydrophobicity and side-chain properties in environment-specific features (Additional file 1: Table 6). Rules generated by this run of the GA had per-class accuracy of 69.0% ± 6.4%. The predictive capacity was lowest for glacier ice (61.6% ± 1.9%) and highest for rock sequences (76.4% ± 3.1%). Rules included 23 of the 64 conserved positions (Fig. 6a). The most common positions were 106 and 72 (Fig. 6b). Certain AAs came up repeatedly in the ruleset, including phenylalanine, alanine, leucine and asparagine. Rules for frozen sediment environments often contained the absence of hydrophobic AAs (F and A) at positions 106 (a-face), 72 and 145 (b-face).

### Feature importance and model interpretation

**Fig. 7:**
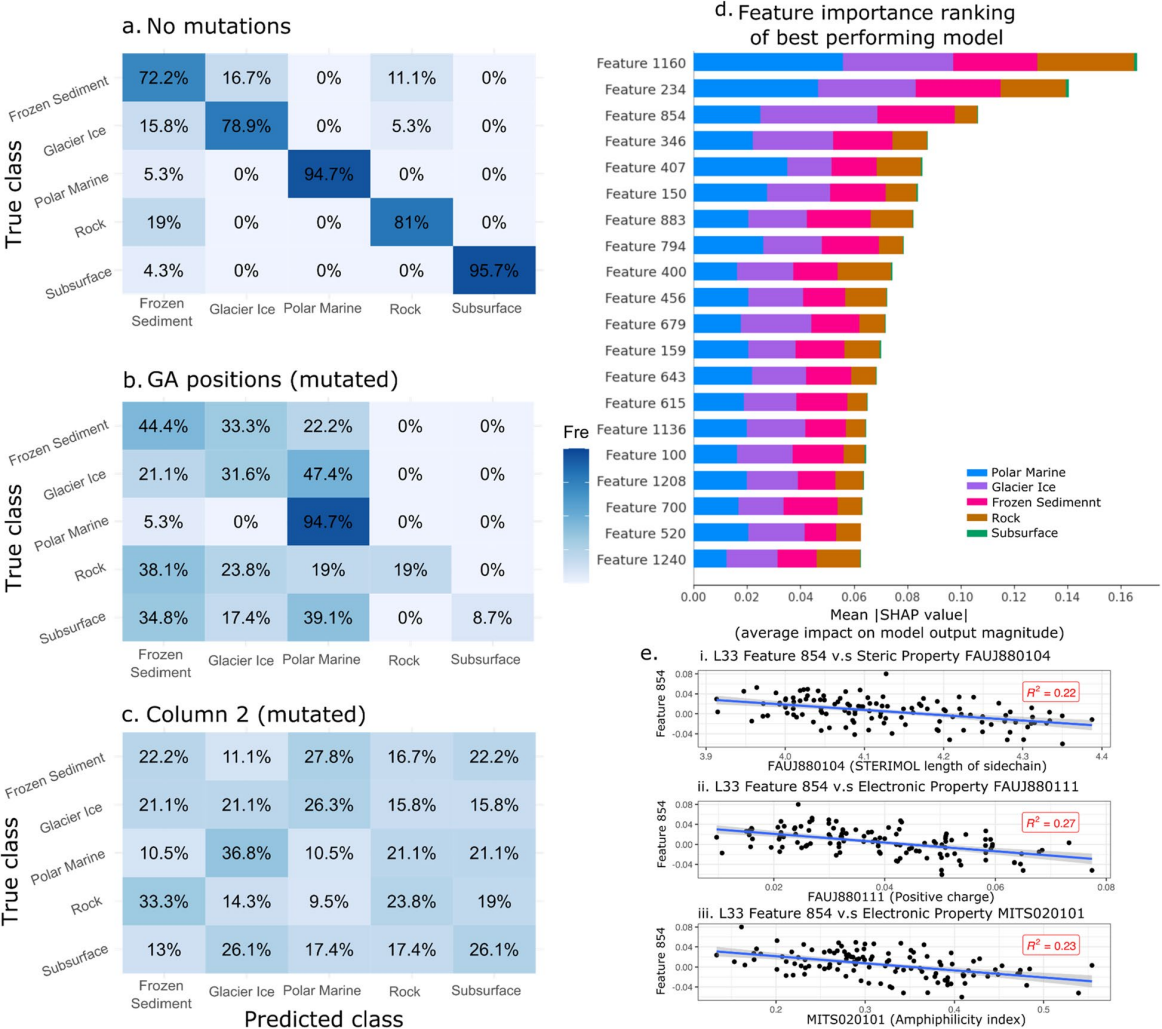
In silico mutagenesis and feature extraction for the best performing artificial neural network (ANN) reveals a column of important residues and a weak correlation with side chain length. **a** Prediction accuracy for the ANN on a subset of 100 test sequences without mutations, for comparison. **b** When all positions identified by the 2-rule genetic algorithm are replaced with random residues, prediction accuracy is reduced, most notably for glacier ice, rock and subsurface. **c** When all positions in Column 2 on the b-face of the protein are replaced with random residues, predictive ability is lost for all environments. **d** Feature importance ranking for best performing ANN using mean SHAP values identifies that L33 features 1160, 234 and 854 are the most important. **e **The strongest association between features and physicochemical properties was Feature 854, which is weakly positively associated with steric properties (i.) FAUJ880104 (STERIMOL length of the side chain); (ii.) FAUJ880111 (positive charge); (iii.) MITS020101 (amphiphilicity index)

In silico mutations of PF11999 DUF3494 sequences revealed an essential role of column 2 of b-face residues in the model’s ability to differentiate between any environments (Fig. 7a). Conversely, changes based on GA rules had an environment-specific effect on model prediction (Fig. 7b). The original prediction accuracy of 84% decreased to 21% when Column 2 was mutated, and the maximum accuracy was 26.1% for any given environment. In silico mutations of residues of other columns were much less impactful (Table 5). When all positions identified by the GA were mutated simultaneously, the model’s ability to classify most environments was reduced, but not to the extent seen with Column 2 alone. Interestingly, polar marine sequences remained 94.7% classifiable even after the GA mutations, suggesting either greater redundancy in predictive features or stronger overall sequence signal in this environment. Phylogenetic structure had a weak effect on model classification correctness where phylum-level classification was possible (McFadden’s *R*^2^ = 0.15), likely due to the associations between environment and phylum described above. 

**Table 5:** Effect of in silico mutations of residues of importance on overall ANN prediction accuracy. Residues of importance identified by the GA with a two-parameter allowance were replaced with random amino acids. All GA residues of importance were mutated, as well as by protein face. Additionally, columns of importance identified through model tuning and sequence comparisons were mutated

Mutated region	Number of residues	Overall accuracy
None	0	84%
All GA residues	22	40%
a-face (GA)	5	81%
b-face (GA)	8	84%
c-face (GA)	4	88%
Between faces	5	86%
Column 2 (b-face)	7	21%
Column 4 (b-face)	7	84%
Column 0 (a-face)	7	79%

When features identified by SHAP (Shapley additive explanations) were correlated with hydrophobic, steric and electronic (HSE) properties of a subset of 100 sequences, weak correlations with steric and electronic properties of the sequence were found (Additional file 1: Table 7). The three most important features for the whole model were features 1160, 234 and 854 (Fig. 7d). Three HSE properties were weakly correlated with feature 854: average side-chain length (*R*^2^ = 0.22), amphiphilicity index (R^2^ = 0.23) and positive charge (*R*^2^ = 0.27). L33 encoding and our ANN are likely capturing complex, higher-order patterns that do not map directly onto single physicochemical properties.

## Discussion

Artificial neural networks (ANNs) are increasingly used to analyse big data in genomics, but have limited interpretability and accessibility. Here, we explored a metagenomics dataset of a taxonomically widespread domain involved in microbial antifreeze activity, using an ANN and a genetic algorithm (GA), which provided additional insights into the biology driving this classification. We obtained a total of 50,669 amino acid sequences of this domain of unknown function (DUF3494) from publicly available metagenome datasets. These sequences were used as input for our feedforward ANN to assess model performance. Depending on the model architecture and hyperparameter optimisation, this ANN was able to classify to identify the environment with good accuracy (75.9% to 97.8%). We also explored a genetic algorithm (GA), which provided additional insights into the biology driving this classification, and it enabled us to understand which features were learned by our ANN.

### Transfer learning

We used transfer learning with the ESM-2 model to build a smaller model that was able to classify PF11999 DUF3494 sequences by their source environment. This model was able to correctly classify a large proportion of sequences, and importantly, our model can do so without making sequence alignments. We then explored diversity and biologically relevant regions of these proteins to examine which residues are driving these environment-associated differences. We were specifically interested in the regions of these proteins which have been suggested to be involved with antifreeze functions. We identified a number of “columns” of amino acids corresponding to protein faces that were of particular importance for ANN learning. Some of these columns were consistent with putative ice-binding sites [34] and/or differences in protein diversity between environments. To complement these analyses, we analysed conserved regions of the sequence with a GA. While the GA could classify sequences less accurately than the ANN, it provided clear rules for classification. To compare both methods, and interpret predictions made by the ANN, we performed in silico mutations on certain residues and examined feature importance. The mutations revealed one column (Column 2 on the b-face) that was of particular importance for the ANN’s predictive capacity. In silico mutations of AAs in this column markedly compromised the predictive ability of the ANN, whilst mutating AAs in other columns had much less impact. Finally, feature importance and subsequent correlation tests revealed additional weak associations between important features and the physicochemical properties of the protein. These results provide a framework for using deep learning methods to gain biological insight into complex protein families.

Our results demonstrate that transfer learning combined with a shallow feedforward ANN is effective in classifying complex biological sequence data. Our aim was to use this method to gain insights into an existing dataset, rather than to test predictive power. Our results support previous studies where two-layer feedforward ANNs (like ours) have been shown to be more effective at classifying “omics data” than alternative architectures [66]. ANN-based sequence classification tasks are often employed to improve on traditional methods such as for taxonomic classification [67] or disease prediction [66]. In the case of our model, however, we not only explored how these sequences can be classified by an ANN, but also how a combination of approaches can improve model transparency and biological interpretability.

Environmental datasets like ours often contain large class imbalances that can be effectively accounted for using class weighting as we have done here [68]. Our model was able to predict sequences from the least abundant environments with comparable accuracy, showing that this approach is well suited to environmental sequence data. Our single-layer model was only marginally less accurate overall than our two-layer model, but it performed less well at classifying lower abundance environments. Possibly, the second layer enabled the model to learn more about these less-represented environments. Transfer learning is an alternative to computing known features of the sequences [69] and it is a particularly promising approach to study non-model systems. Our results support previous research that ESM-2 is an effective, accessible method for learning diverse features of protein sequences [15, 55], especially when extensive prior information is not available.

### Genetic algorithm (GA)

The GA is an effective tool to complement an ANN. Firstly, the GA can be used as a “hypothesis generator” for in silico mutagenesis approaches. The GA can help identify residues that are likely to be more important for ANN learning, reducing the targets explored by in silico mutagenesis, which can be time-consuming [70]. We found that mutating the residues that had been identified as important by the GA had a meaningful impact on the ANN’s predictive capacity. Using prior biological knowledge about the sequences to identify and mutate columns of interest also successfully identified important residues. However, this approach is limited to well-studied model proteins. The GA also successfully identified residues which had an effect on environmental classification, but it did so *a priori* without information. ESM-2 encoding contains information about residue-residue contacts and physicochemical features [9] information which the GA did not have as input. In addition, the GA was able to provide more interpretable results. Compared to the ANN, which showed very strong effects of mutating structurally local residues (i.e. columns), GA rules were spread across the protein.

### Environmental adaptation of DUF3494 ice-binding proteins

A combination of vertical inheritance constraints (i.e. a phylogenetic signal) and functional adaptation may be driving the observed differences in PF11999 DUF3494 sequences between environments. We detected a moderately strong association between environment and phylum (*R*^2^ = 0.48), which may be a sign that evolutionary history constrains DUF3494 diversity, as is found elsewhere [38]. For example, 42% of Rock sequences were classified as being Actinomycetota, supporting a possible association with taxonomy. We found evidence of convergence between polar marine and glacier ice in functionally important columns of the domain (Fig. 5). This is noteworthy because there is little taxonomic overlap between both communities, and there is not always a strong association between environment and phylum. While glacier ice and sea ice are very different as substrates for microbial life [71] they may converge at the ice-microstructure scale, as both can contain high abundances of pure ice with larger crystal sizes [72, 73]. Conversely, frozen sedimentary and subsurface environments have lower ice content [74] with high levels of humic substances and sediment. Large amounts of these impurities shape ice microstructure and result in smaller ice grains with higher nucleation rates [75]. It is possible that an abundance of smaller ice grains rather than large crystals may select for different functions of DUF3494 ice binding (discussed below) [76]; however, whether this would result in higher diversity in functionally important columns is unclear (see Additional File 6 for further discussion of the environmental adaptation of DUF3494 sequences)[77–79].

### Molecular biology of DUF3494

Feature importance and in silico mutagenesis identified environment-specific variation in DUF3494 sequences which may inform us about the evolution of protein family 11999 (ice-binding like). Examining these sequences at the level of environment specificity determined by the ANN allowed us to distinguish that polar marine and glacier ice often show the same patterns down 2 columns of importance. In silico mutagenesis then revealed that the first column (b-face column 2) was essential for distinguishing between all environments. This column has been implicated in antifreeze efficiency in in vitro studies of DUF3494 proteins [34]. The consistency of small hydrophobic alanines down this column in these environments is thought to form a “trough” which contributes to ice-binding [80]. Mutations of alanine residues to bulkier side-chain AAs in this column of the protein have specifically been shown to diminish ice-binding activity [34, 80]. In less pure ice environments (frozen sediment, subsurface, rock) hydrophilic AAs serine, glycine and threonine are more dominant down the column, possibly implying lower ice-binding efficiency in these environments. Ice-binding protein activity is usually quantified in terms of ice-recrystallisation inhibition (IRI) and thermal hysteresis (TH). In the former, the formation of large ice crystals is inhibited in favour of smaller, less damaging crystals, while in the latter, the freezing point of ice is depressed [25]. DUF3494 proteins display a diverse range of these functions; however, a structure–function relationship has not been systematically investigated [77, 81–83]. A well characterised ice-adhesin IBP from the Antarctic marine bacterium *Shewanella frigidimarina* (*Sf*IBP) contains only alanines down column 2 consistent with our observation in polar marine environments and has been shown to have high IRI and hyperactive TH activity [84]. Conversely, the DUF3494 from symbiotic bacteria associated with the polar sedimentary ciliate *Euplotes focardii* (*Efc*IBP) contains serine, threonine and alanine in this column [35]. This protein is 40 × less effective at IRI and slightly less effective at TH compared to the *Sf*IBP and displayed atypical behaviour when exposed to ice compared to other IBPs [84]. Through our ANN classification and in silico mutagenesis we have observed that this may be a more general ecological trend. 

## Conclusions

This study demonstrates the successful application of an ANN and GA to classify PF11999 DUF3494 protein sequences by environment. The ANN’s environment-specific predictions were complemented by the GA, which identified critical residues and sequence patterns. Together, these methods revealed sequence features that may drive environmental specificity, offering a novel approach to understanding DUF3494 protein diversity. The GA also served as an effective hypothesis generator for in silico mutagenesis, improving interpretability by identifying residues of importance. This combined approach highlights the potential of integrating GAs as pre-training tasks in ANNs, broadening their application in protein classification and evolutionary studies. 

## Supplementary Information

Additional file 1: Table 1: Search terms used to find appropriate datasets for this study. Search terms included all combinations of the first term and second term columns.

Additional file 2: Supplementary Methods.

Additional file 3: Results.

Additional file 4: Supplementary Figures.

Additional file 5: Results.

Additional file 6: Supplementary discussion.

## Data Availability

This manuscript used publicly available metagenomics datasets, whose NCBI accessions are available in Additional file 1: Table 2. Sequences for the MOSAiC samples used is available at 10.6084/m9.figshare.25765707.v1 [85]. Code for the bioinformatics pipeline, data preprocessing and artificial neural network (ANN) can be found at https://github.com/jcwinder/Deep-learning-insights-into-ice-binding-protein-ecology (10.5281/zenodo.16266611) [86]. Processed datasets used for building the model are also on the GitHub.
