# Supplementary material for: Environmental adaptations in metagenomes revealed by deep learning
Source: BMC Biol. 2025 Aug 11;23:252. doi: 10.1186/s12915-025-02361-1 (PMC12337378; doi:10.1186/s12915-025-02361-1)
Supplement: Supplementary file 1 — Additional file 1: Table 1: Search terms used to find appropriate datasets for this study. Search terms included all combinations of the first term and second term columns. [file 12915_2025_2361_MOESM1_ESM.pdf]

Additional File 1: Table 1: Search terms used to find appropriate datasets for this study. Search terms included all combinations of the first term and second term columns.

	First term (one of)		Second term (one of)
1	ice	+	Metagenome /
2	microbial mat		
3	permafrost		
4	glacier		
5	rock		
6	cave		
7	snottite		
8	biofilm		
9	natural biofilm		Metagenomics /
10	polar		
11	hydrothermal		
12	hydrothermal vent		
13	hot spring		
14	biocrust		
15	surface		
16	endolith		
17	deep subsurface		Microbiome /
18	subsurface		
19	rock varnish		
20	desert		
21	sand		
22	artificial biofilm		
23	plastic		
24	free biofilm		
25	stromatolite		
26	mine		Biofilm
27	oral		
28	dental plaque		
29	tooth		
30	saliva		
31	leaf biofilm		
32	epiphytic		
33	phycosphere		
34	phyllosphere		

35	rhizosphere		

Additional File 1: Table 2: NCBI Bioprojects from which sequences were obtained for this project

Accession
PRJNA813429
PRJNA445613
PRJNA360211
PRJEB12327
PRJNA588686
PRJNA596250
PRJNA266338
PRJNA630822
PRJDB11497
PRJNA552298
PRJNA88155
PRJNA528687
PRJNA893676
PRJNA657180
PRJNA506221
PRJNA734336
PRJNA798087
PRJNA673486
PRJEB47746
PRJNA223407
SRP093781
PRJNA266334
PRJNA394028
PRJNA261849
PRJEB30872
PRJNA601698
PRJNA505516
PRJEB42267
PRJEB47746
PRJNA825327
PRJNA642310
PRJNA485054
PRJNA777757
PRJEB38431
PRJNA616285
SRP006444
PRJNA188465
PRJEB12283
PRJNA741999
PRJNA193416
PRJNA911846
PRJNA694468
PRJNA848445
PRJNA646106
PRJNA340462
SRR1636513,
SRR2058407,
SRR1636510,

SRR2058405
SRR1636516,
SRR1636517
SRR1636508,
SRR1636509
PRJEB7866
PRJEB9204
PRJNA335670
PRJNA336655
PRJNA563887
PRJNA967693
PRJNA234377
PRJNA640378
PRJNA540505
PRJNA663466
PRJNA260488
PRJNA513359-376
PRJNA899190-222
PRJNA899243-258
PRJNA899266-PRJNA899294,
PRJNA905198
PRJNA285514
PRJEB48900
PRJNA290687
PRJEB21768
PRJNA679196
PRJNA623622
PRJNA265986
PRJNA540872
PRJEB13142
PRJNA276301-306
PRJNA257561
PRJNA578106
PRJNA259227
PRJNA309376
PRJNA495084
PRJNA755678
PRJNA753157
PRJNA556392
PRJEB36085
PRJNA438021
PRJEB15404
PRJNA438384
PRJNA761511
PRJNA438384
PRJNA481022
PRJNA669531
PRJEB37023
PRJNA643293
PRJNA317551
PRJNA800583
PRJNA555798
PRJNA624048
PRJNA675914
PRJNA438021
PRJNA319196
SRR15275520
PRJNA728043

ERR3150493,
ERR3150492
PRJNA362455
PRJNA725617
PRJNA833075
PRJNA547717
SRR3100146-155
PRJEB38470
PRJEB30524
PRJNA674450
PRJNA645385
PRJNA645371
PRJNA766357
PRJNA255922
PRJNA528558
PRJNA625082
PRJNA230363
PRJEB43389
PRJEB67641
PRJEB30331
PRJEB31185
PRJNA383868
PRJNA78025
PRJNA289925
PRJEB6997
PRJEB24090
PRJNA380727
PRJNA396840
PRJEB14383
PRJNA217052
PRJNA188481

Additional File 1: Table 3: Model architectures for three models with L33 encoding.

Differences between models were: the number of environments, number of training epochs, learning rate and batch size. Note that where two hidden layers were tested, one hidden layer was always also tested.

Number of environments	Epochs	Learning rate	Batch size	Number of hidden layers	Hidden nodes	Loss/Accuracy
11	100	0.001	256	1	800	0.898/0.664
			512	1	800	0.940/0.637
			1024	1	800	1.03/0.599
		0.0001	256	1	800	1.34/0.470
			512	1	800	1.38/0.431
			1024	1	800	1.31/0.413
	500	0.001	256	1	800	0.993/0.770
			512	1	800	1.05/0.706
		0.0001	256	1	800	0.927/0.628
			512	1	800	1.03/0.613
8	100	0.001	256	1	800	0.377/0.875
			512	1	800	0.381/0.875
		0.0001	256	1	800	1.05/0.686
			512	1	800	1.13/0.671
	500	0.001	256	1	800	0.447/0.922
			512	1	800	0.406/0.923
		0.0001	256	1	800	0.800/0.743
			512	1	800	0.864/0.725
5	100	0.001	256	1	800	0.334/0.899
				2	800/400	0.362/0.911
			512	2	800/400	0.315/0.920
		0.0001	256	2	800/400	0.278/0.907
			512	2	800/400	0.321/0.891
	200	0.001	256	2	800/400	0.465/0.925
			512	2	800/400	0.410/0.926
		0.0001	256	2	800/400	0.278/0.920
			512	2	800/400	0.271/0.916
	500	0.001	256	1	800	0.443/0.928
				2	800/400	0.628/0.926
			512	1	800	0.392/0.924
				2	800/400	0.345/0.925
		0.0001	256	1	800	0.265/0.918
				2	800/400	0.398/0.930
			512	1	800	0.267/0.913
				2	800/400	0.352/0.925

Table 4:

	Positional Multinomial logistic regression		
Class	Precision	Recall	F1 score
0: Frozen sediment	0.6093	0.6782	0.6419
1: Rock	0.8833	0.8544	0.8686
2: Subsurface	0.9255	0.8474	0.8848
3: Polar marine	0.9297	0.9581	0.9437
4: Glacier ice	0.7148	0.6961	0.7053

	L33 Multinomial logistic regression		
Class	Precision	Recall	F1 score
0: Frozen sediment	0.5937	0.6312	0.6119
1: Rock	0.8308	0.8548	0.8426
2: Subsurface	0.8468	0.8295	0.8381
3: Polar marine	0.8769	0.9107	0.8935
4: Glacier ice	0.7430	0.6610	0.6996

	HMM assignment		
Class	Precision	Recall	F1 score
0: Frozen sediment	0.48	0.15	0.23
1: Rock	0.57	0.89	0.69
2: Subsurface	0.04	0.59	0.07
3: Polar marine	0.99	0.29	0.45
4: Glacier ice	0.13	0.57	0.21

Table 5: 20-rule GA ruleset

Target	Totalpc	PredTerms	PredVariables	PredExpression
Frozen_Sediment	69.36%	10	106 109 145 71 72 77 82	((71 <> F & 82 <> W) & (72 <> F & (109 <> V & (145 <> A | (145 <> A & ((109 <> I | (77 = A & 109 <> I)))) | 106 <> F))))
Frozen_Sediment	67.86%	6	106 107 145 71 81 83	(((145 <> A | 106 = Y) & 107 <> L)) | (83 = C | (81 = L | 71 = X))
Frozen_Sediment	67.31%	6	145 71 78 81 83	(78 = A | ((71 <> P & ((81 = C | 78 = H)) | 83 = C) | 145 <> A))
Frozen_Sediment	69.23%	10	106 108 132 134 71 72 80 83 97	((134 <> E & 132 = G) | (106 <> F | (132 = D | 72 = G) | (97 = N & 108 = F))) | ((83 = C | ((80 = H | 71 = K))))
Frozen_Sediment	67.82%	4	106 145 153 97	(145 <> A & (153 <> L & (97 <> D | 106 = W)))
Frozen_Sediment	67.80%	4	106 108 72 77	(108 <> I & ((77 = L | 106 <> F) & 72 <> F))
Frozen_Sediment	65.77%	6	106 145 71 72 81	(145 <> A | ((81 = S | (106 <> F & (71 <> Y | 72 = E) | 81 = L))))
Frozen_Sediment	66.72%	3	145 153 71	((153 = D | 145 <> A) & 71 <> V)
Frozen_Sediment	65.63%	5	108 143 153 77 83	(83 = L | (153 <> L & 108 = F) & ((143 <> T & 77 <> F)))
Frozen_Sediment	65.35%	5	106 133 72 81 97	(106 = W | (81 = K | (72 = H | (97 <> D | 133 = T))))
Glacier Ice	68.75%	9	105 125 126 152 171 173 20 30 73	((30 = Y | (126 <> W & 173 <> A))) | (73 = D | ((20 = W | (105 = G | (125 = N | 171 = G)) | 152 = Q)))
Glacier Ice	66.87%	11	106 125 135 144 72 73 78 95	(78 = N | ((72 = G | (95 = Q | (73 = D | (106 <> F | 78 = K)) | 144 = A) & (135 <> Y & 125 <> D))) | (((73 = N | 73 = Q))))
Glacier Ice	65.36%	10	105 106 107 126 173 71 73 82 97	(97 = L | (173 <> A | 126 = X) | (73 = H | 107 = Y)) | ((106 <> F & ((105 = L | ((82 = Y | 105 = L))) | 71 = C)))
Glacier Ice	65.39%	7	106 143 173 72 97	((97 <> Y & (72 <> I & 173 <> A))) | (106 = W & ((143 <> A & 72 <> I) | 143 = I))
Glacier Ice	63.95%	9	106 107 126 135 144 73 95 97	(((73 = I | (135 = I | 106 <> F) | (95 = V | (((73 = I | 107 = I) | 126 = P) & 97 <> S) & 144 <> G))))
Glacier Ice	64.10%	4	106 107 173 78	(78 = N | (107 <> L & (173 = S | 106 = W)))
Glacier Ice	62.60%	8	105 106 126 133 142 144 76	((142 = A | (133 <> G & (106 <> F & 76 <> N))) & (105 <> D | (126 = W & (142 = A & 144 <> S))))
Glacier Ice	61.06%	8	106 124 127 133 151 171 71	((171 = G | (133 = V & ((106 = W | 71 = Y)) & 127 <> C)) & (133 <> X | (151 <> E & 124 <> Q)))
Glacier Ice	62.57%	7	105 106 107 133 154 171 80	((171 = G & 107 <> H)) | (80 <> V & (105 = T | (133 = V & (154 <> D & 106 <> F))))
Glacier Ice	61.45%	11	105 106 124 127 133 71 78 96	((106 = W | ((133 <> L & 78 = K)) | (((127 = A | (127 = A | 105 = T)) | (127 = A | (124 = A | 127 = A)) | 71 = W) | 96 = C)))
Polar Marine	83.21%	9	134 145 71 72 94 95 96	((134 = L | ((94 = I | (145 = A & 72 <> L)) | ((72 = F | 71 = V) | 95 = H) | (96 <> L & 72 <> L))))
Polar Marine	80.76%	7	105 133 145 71 72 94	(94 = I | (71 = W | (105 = I | ((133 = D | (94 = D | 145 = A))) & 72 <> L)))
Polar Marine	80.34%	5	152 71 72 77	(71 <> D & ((72 <> G & (72 <> L | 77 = M)) | 152 = I))
Polar Marine	79.23%	5	108 145 71 72 80	(145 = A | (80 <> H & (71 = V | (72 = F | 108 = I))))
Polar Marine	78.95%	10	108 142 145 71 77 79 95	(71 <> D & (((142 = T & 142 <> T)) | (95 = Y | 77 = M) | (71 = V | ((79 = T | 71 = S) | (145 = A | 108 <> F)))))
Polar Marine	77.83%	5	133 134 143 144 72	((134 = I | ((144 = V | 133 = I) & 143 = V) | 72 <> L))
Polar Marine	77.48%	6	108 134 143 152 71 72	((71 <> X & (72 <> L | (108 = M | (143 = T | 134 =

<table>
<tr><td></td><td></td><td></td><td></td><td>I) | 152 = I))))</td></tr>
<tr><td>Polar Marine</td><td>77.05%</td><td>4</td><td>143 71 72 81</td><td>((71 = A | 81 <> S) & (143 = V | 72 <> L))</td></tr>
<tr><td>Polar Marine</td><td>76.26%</td><td>9</td><td>105 133 145 71 80 95</td><td>((71 = V | (133 = G | (145 = A | (105 = I & ((80 = G & 95 <> W))) & (80 = G | 80 = G))) | 95 = L))</td></tr>
<tr><td>Polar Marine</td><td>76.06%</td><td>4</td><td>109 142 145 82</td><td>((145 = A | (109 <> Q & 142 <> T) & 82 <> F))</td></tr>
<tr><td></td><td></td><td></td><td></td><td></td></tr>
<tr><td>Rock</td><td>81.82%</td><td>5</td><td>10 132 133 173 8</td><td>(((173 = A & 8 <> A) | 132 = L) & (133 = G | 10 = T))</td></tr>
<tr><td>Rock</td><td>81.41%</td><td>6</td><td>10 132 71 72 80 82</td><td>((10 = T | 71 = P) & ((72 <> F | 132 = E)) | (82 = T | 80 = H))</td></tr>
<tr><td>Rock</td><td>81.01%</td><td>9</td><td>106 109 124 133 71 72 78</td><td>((72 = L | (72 = L | (72 = L | (71 = D & ((124 <> W & 133 <> E) | 109 = P)))) & (106 = F | 78 = V)))</td></tr>
<tr><td>Rock</td><td>78.99%</td><td>8</td><td>107 132 134 71 72 96</td><td>(134 <> L & (134 <> T & (96 = L & ((107 = F | 72 = L))) | 71 = D) | ((71 = D | 132 = A)))</td></tr>
<tr><td>Rock</td><td>79.23%</td><td>8</td><td>106 132 134 72 81</td><td>((132 = D | (106 = F | 132 = E) & (((134 <> L & 72 = L)) | 81 = G) | (81 = G | 72 = G)))</td></tr>
<tr><td>Rock</td><td>77.05%</td><td>5</td><td>133 71 72 81 82</td><td>(71 = D | ((81 = V & (133 <> I | 82 = C)) & 72 <> F))</td></tr>
<tr><td>Rock</td><td>77.29%</td><td>4</td><td>106 124 132 72</td><td>(124 = Y | (106 <> W & (72 = L | 132 = X)))</td></tr>
<tr><td>Rock</td><td>75.57%</td><td>6</td><td>106 108 132 71</td><td>((71 = P | (71 = D & (108 <> M & 106 = F)) & (132 <> M & 108 <> M)))</td></tr>
<tr><td>Rock</td><td>76.05%</td><td>6</td><td>134 142 71 79</td><td>((((71 <> S & 142 = T) & 134 <> W) & (71 = D | 79 = A) & 142 = T))</td></tr>
<tr><td>Rock</td><td>74.57%</td><td>7</td><td>107 109 134 135 71 77 97</td><td>(135 <> W | ((71 = D & (109 <> R | (97 <> E & 77 = E)))) & (107 <> K & 134 <> W))</td></tr>
<tr><td></td><td></td><td></td><td></td><td></td></tr>
<tr><td>Subsurface</td><td>78.02%</td><td>9</td><td>144 170 19 5 71 95</td><td>((5 <> G & (170 <> C & 19 <> T))) & (95 <> F & ((19 <> L | (71 = I | 144 = D)) & (95 <> V & 95 <> Y)))</td></tr>
<tr><td>Subsurface</td><td>77.01%</td><td>8</td><td>134 19 26 5 71 95</td><td>(((26 <> A | ((19 = I & 71 <> L)) & (71 <> V & 134 <> L)) | 5 = E) & (71 <> A | 95 = Q))</td></tr>
<tr><td>Subsurface</td><td>76.31%</td><td>20</td><td>107 125 27 73 77 78 80 95</td><td>((95 <> V & (78 <> R & (((73 <> C & 95 <> N)) & (80 <> N & (78 <> E & ((78 <> F & (107 <> Y & (78 <> G & 107 <> F))) & (73 <> P & (107 <> T & ((27 <> V | 125 = T))))) & (78 <> H & (77 = L & 78 <> Y) & 107 <> L))))) & (78 <> K & 78 <> P)))</td></tr>
<tr><td>Subsurface</td><td>72.41%</td><td>8</td><td>124 152 26 27</td><td>(26 = Y | (((26 = L | (124 = R | (124 = M | (26 = T | 27 <> V)) & 152 <> T)) | 26 = S)))</td></tr>
<tr><td>Subsurface</td><td>74.45%</td><td>7</td><td>106 108 154 173 177 79</td><td>(((108 <> I & (154 <> I & 79 <> A)) | 177 = S) & (79 <> D & (173 <> S | 106 = W)))</td></tr>
<tr><td>Subsurface</td><td>72.54%</td><td>10</td><td>105 106 142 72 81 94 95 97</td><td>((((81 = C | 105 = T) | (142 = A | (94 = A | 106 = C) | 142 = D) | 81 = L)) | (((95 <> Y & 97 <> N) & 72 = L)))</td></tr>
<tr><td>Subsurface</td><td>70.17%</td><td>8</td><td>108 125 134 145 71 79 83</td><td>(108 <> M & (83 = Q | (71 = D | (134 = K | 79 <> A))) & ((125 = S | (145 <> A & 79 <> E))))</td></tr>
<tr><td>Subsurface</td><td>69.73%</td><td>7</td><td>125 144 172 173 80</td><td>(173 = A | ((144 = L | ((125 = A | (80 = H & 172 <> M))) | 144 = E) & 172 <> F))</td></tr>
<tr><td>Subsurface</td><td>68.18%</td><td>8</td><td>107 152 173 78 80 96</td><td>(107 = D | ((80 = H | ((173 = A & 78 <> K)) & 96 <> I) & 96 <> F) | ((152 = I | 152 = N)))</td></tr>
<tr><td>Subsurface</td><td>70.60%</td><td>7</td><td>145 178 78 97</td><td>(78 <> S & (97 = S | ((145 = T & 178 <> I)) & (78 <> K & (178 <> C & 78 <> R))))</td></tr>
</table>

Table 6: 2-rule GA dataset

Target	Totalpc	PredTerms	PredVariables	PredExpression
Frozen_Sediment	65.31%	2	106 145	(145 <> A | 106 <> F)
Frozen_Sediment	65.34%	2	72 81	(81 <> V | 72 = L)
Frozen_Sediment	65.38%	1	145	145 <> A
Frozen_Sediment	64.96%	1	145	145 <> A
Frozen_Sediment	60.83%	2	72 97	(97 <> D | 72 <> F)
Frozen_Sediment	63.70%	2	106 72	(72 <> F & 106 <> F)
Frozen_Sediment	63.51%	2	106 72	(106 <> F | 72 = P)
Frozen_Sediment	60.98%	1	72	72 <> F
Frozen_Sediment	63.76%	2	81 97	(97 <> D | 81 = L)
Frozen_Sediment	61.17%	2	106 72	(72 = P | 106 = W)
Glacier Ice	63.32%	2	106 173	(106 = W | 173 <> A)
Glacier Ice	64.31%	2	173 18	(173 <> A | 18 = S)
Glacier Ice	62.32%	2	106 173	(106 <> F | 173 <> A)
Glacier Ice	63.20%	2	143 173	(173 <> A & 143 <> V)
Glacier Ice	62.55%	2	173 81	(173 <> A | 81 = L)
Glacier Ice	61.92%	2	106 171	(171 = G | 106 <> F)
Glacier Ice	60.00%	2	106 126	(106 <> F | 126 = H)
Glacier Ice	60.17%	2	106 152	(106 <> F | 152 = V)
Glacier Ice	59.01%	2	106 94	(94 <> I & 106 = W)
Glacier Ice	59.35%	2	106 80	(106 = W | 80 = A)
Polar Marine	77.70%	2	71 72	(71 <> D & 72 <> L)
Polar Marine	76.94%	2	145 72	(145 = A | 72 = F)
Polar Marine	76.72%	2	145 72	(72 = F | 145 = A)
Polar Marine	75.34%	2	71 72	(72 <> L | 71 = C)
Polar Marine	74.80%	2	145 71	(145 = A & 71 <> D)
Polar Marine	74.72%	2	145 71	(71 <> D & 145 = A)
Polar Marine	74.92%	1	72	72 <> L
Polar Marine	74.89%	1	72	72 <> L
Polar Marine	74.41%	2	145 71	(145 <> T & 71 <> D)
Polar Marine	72.24%	1	145	145 = A
Rock	80.04%	2	10 72	(10 = T & 72 = L)
Rock	79.76%	2	10 72	(72 = L & 10 = T)
Rock	79.62%	2	10 142	(10 = T & 142 = T)
Rock	79.42%	2	10 72	(10 = T & 72 = L)
Rock	75.92%	2	106 145	(145 <> A & 106 = F)
Rock	74.88%	2	106 71	(71 = D & 106 = F)
Rock	74.75%	1	72	72 = L
Rock	74.72%	1	72	72 = L
Rock	72.26%	2	142 71	(71 = D & 142 = T)
Rock	72.46%	1	71	71 = D
Subsurface	73.15%	2	152 5	(5 <> G & 152 <> T)
Subsurface	70.16%	2	152 72	(72 <> M & 152 <> T)
Subsurface	69.52%	2	145 152	(152 <> T & 145 = T)
Subsurface	66.53%	2	153 5	(153 <> L & 5 <> G)
Subsurface	68.36%	2	152 77	(152 <> T & 77 = L)
Subsurface	66.15%	2	145 78	(78 <> K & 145 <> A)
Subsurface	68.03%	2	7 77	(77 = L & 7 = I)
Subsurface	66.75%	2	173 78	(78 = A | 173 = A)
Subsurface	65.82%	2	106 153	(106 = W | 153 = I)
Subsurface	66.75%	2	153 178	(153 <> L & 178 = V)

Additional File 1: Table 7: Hydrophobic, steric and electronic properties of DUF3494 sequences correlated with features of ESM-2 encoded sequences. HSE properties were calculated for the whole proteins and by protein region. Weak correlations were identified, this table contains correlations which had an R^2 above 0.1.

Feature	Grouping	Variable		
1160	Hydrophobic	FASG760102	Melting point	0.16
	Electronic	MITS020101	Amphiphilicity index	0.12
854	Steric	CHOC750101	Average volume of buried residue	0.16
		CHOC760101	Residue accessible surface area in tripeptide	0.18
		FAUJ880101	Graph shape index	0.12
		FAUJ880103	Normalized van der Waals volume	0.18
		FAUJ880104	STERIMOL length of the side chain	0.22
		FAUJ880106	STERIMOL maximum width of the side chain	0.16
		JANJ780101	Average accessible surface area	0.21
		LEVM760102	Distance between Cα and centroid of side chain	0.18
		LEVM760103	Side chain angle theta(AAR)	0.13
		LEVM760104	Side chain torsion angle phi	0.13
		LEVM760105	Radius of gyration of side chain	0.18
	Hydrophobic	HOPA770101	Hydration number	0.12
	Electronic	CHAM830107	A parameter of charge transfer capability	0.11
		FAUJ880107	N.m.r. chemical shift of alpha-carbon	0.12
		FAUJ880111	Positive charge	0.27
		MITS020101	Amphiphilicity index	0.23
