# Supplementary material for: Environmental adaptations in metagenomes revealed by deep learning
Source: BMC Biol. 2025 Aug 11;23:252. doi: 10.1186/s12915-025-02361-1 (PMC12337378; doi:10.1186/s12915-025-02361-1)
Supplement: Supplementary file 2 — Additional file 2: Supplementary Methods. [file 12915_2025_2361_MOESM2_ESM.pdf]

Additional File 2: Supplementary Methods

1. Artificial neural network architecture

1.2 Loss function:

ANNs learn by adjusting the weights in the hidden layer to optimise performance. Internally in the model, performance is measured using a loss function. The loss function is an error function which represents the difference between the model output and the goal output. The higher the loss, the worse the model's predictions are. The type of loss function used is dependent on the type of output. A categorical crossentropy loss function was used for multiclass classification. Crossentropy loss functions, or log loss functions, take the softmax output of predicted probability distribution across classes for a given input, and compare them to the actual probability distribution which would contain "1" for the correct class and "0" for all other classes. The loss function measures the log of the difference between the probability distributions. The use of a logarithmic loss function penalises heavily for models which are confidently wrong. For example, if the actual label is 1, if the predicted label is closer to 0, the loss is exponentially higher.

When working with imbalanced datasets - where some classes are much more common than others - class weighting can be applied to the loss function. This assigns a higher penalty to mistakes made on under-represented classes, helping to prevent the model from becoming biased toward the majority classes. In practice, this involves multiplying the loss contribution from each class by a weight inversely proportional to its frequency in the dataset.

2. Testing environment specificity levels

2.1 Removing environments from the model

Environments were not included in Model 3 because of three things: a. abundance was too low to determine whether there was simply not enough data for the model to learn a classification, b.

they could not be classified by the model regardless of optimisation and c. misclassification patterns did not imply a scientifically meaningful grouping.

3. Hyperparameter testing

The data was split for training, validation and testing in two phases. First, the data was split into 80% training and 20% test using a stratified shuffle split to ensure random sampling proportional to class abundance (i.e that a representative subset of each environment type was selected). The test data was set aside to assess model performance after training. Training data was used for the model to learn features of sequences and update weights based on performance. K-fold cross-validation (kfcv) was used to train the model. During kfcv, the data is split into *k* random groups. The model fit is then looped over these *k* groups. For each iteration of the loop, the group *k* is held aside as the validation set, and the model is fit using the remaining training set. The model is discarded and its evaluation score is kept. This method reduces potential bias in the training/validation split. Five-fold cross validation (i.e *k*=5) was used for this model.

Hyperparameters are static features of the model which are decided before the model is run, which influence its learning behaviour [1]. Hyperparameter values can therefore influence model success, and they should be optimised for the best predictive capacity of a model. Hyperparameters which we tested were: number of training epochs, batch size, and learning rate.

Number of epochs determines the number of times the entire dataset is passed forward and backward through the ANN during the training process [2]. Each pass through the entire dataset is called an epoch. Increasing the number of epochs allows the model to potentially learn more complex patterns in the data but may also increase the risk of overfitting if not controlled properly. We assessed overfitting via the training/validation loss and accuracy curves for each

model. Overfitting was said to be occurring when validation loss increased while training loss remained stable, and accuracy continued to increase or did not change.

Batch size is the number of training examples utilised in one iteration [3]. In each iteration, the model processes a batch of training samples, computes the gradients, and updates the model's parameters. A larger batch size can lead to faster training as it exploits parallelism in hardware better, but it requires more memory. Conversely, a smaller batch size may lead to slower convergence but can provide more noise resilience, and it allows for better generalisability.

The learning rate determines the step size at each iteration while moving toward a minimum of the loss function [3]. It controls the magnitude of parameter updates during training. A higher learning rate allows the model to learn faster, but it may also cause instability or overshooting. Instability here refers to the reproducibility of results, while overshooting refers to skipping over the minimum of the loss function, resulting in oscillations. Conversely, a lower learning rate ensures more stable convergence but may lead to slower training.

Four models were run with different levels of environment specificity as outlined in Table 1. For each of these, a number of hyperparameter combinations were tested (Additional File 1: Table 3). The differences in the number of hyperparameter combinations tested for each model are due to: computational intensity and filtering of hyperparameter combinations which lead to poor results in the first set of models.

<u>Formula:</u> Shannon diversity

$$H = -\Sigma p_i * ln(p_i)$$

Where:
H = Shannon diversity
p_i = the proportion of the entire community made up of species (amino acid) i

Example: 3 sequences:

1. GHI
2. GLI
3. GEL

At position 1, H = -((1 * 0)) = 0
At position 2, H = -((⅓ * ln(⅓)) + (⅓ * ln(⅓)) + (⅓ *ln(⅓))) = 1.10
At position 3, H = -((⅔ * ln(⅔)) + (⅓ * ln(⅓))) = 0.64

12. Model interpretation

12.1 in silico *mutant generation*

We also constructed *in silico* mutant sequences, randomly replacing amino acids based on residues of importance identified with the GA. The goal with this was to identify if different regions of the protein structure were more important to the ability of the ANN to classify sequences. First, a subsample of 100 DUF3494 sequences (stratified, with equal proportions) was isolated from the test dataset (unseen by the model, not used for training/validation). Each of these full sequences was mapped back onto its respective alignment, as the GA refers to the aligned position. Positions of importance were identified by the 2-rule iteration of the GA. These locations were separated by their position on the 3D structure of the protein. We ran five iterations of a random replacement function, for each of: positions of importance (PoI) on the a-face, PoI on the b-face, PoI on the c-face, PoI between faces and for all PoI. This function replaced the amino acid at each position of importance for that iteration with a randomly selected amino acid. The resulting sequences were then mapped back into the original full-length sequence. The full-length *in silico* mutants were then run through the L33 encoding process as described above. The ANN was then used to predict the environment of each of these new sets of sequences, as well as their original counterparts. The accuracy and misclassification pattern of these predictions was then used to identify whether a given region of the protein had a larger impact on the predictive ability of the model.

References

1. Yang L, Shami A. On hyperparameter optimization of machine learning algorithms: Theory and practice. Neurocomputing. 2020;415:295–316. https://doi.org/10.1016/j.neucom.2020.07.061.

2. Xu Y, Qian Q, Li H, Jin R. Why Does Multi-Epoch Training Help? 2021. https://doi.org/10.48550/arXiv.2105.06015.

3. Smith SL, Kindermans P-J, Ying C, Le QV. Don’t Decay the Learning Rate, Increase the Batch Size. 2018. https://doi.org/10.48550/arXiv.1711.00489.
