# Supplementary material for: Environmental adaptations in metagenomes revealed by deep learning
Source: BMC Biol. 2025 Aug 11;23:252. doi: 10.1186/s12915-025-02361-1 (PMC12337378; doi:10.1186/s12915-025-02361-1)
Supplement: Supplementary file 3 — Additional file 3: Results. [file 12915_2025_2361_MOESM3_ESM.pdf]

Additional File 3: Results

1.Benchmarking:

In addition to the LR-PI and HMM approaches described in the main text, we built logistic regression approach based on L33 encoded sequences (LR-L33). This model had an average per-environment accuracy of 77.8% ± 12.4% on the test dataset, with lowest accuracy for frozen sediment (63.12%) and highest for polar marine (91.07%) (Figure 1).

Our ANN can also be applied to classify groups of sequences which have limited homology, compared to the alignment-based approaches described above. To demonstrate this, we ran a version of our model which included PF20597 - whose DUF3494 shares structural, but not sequence, similarities with that of PF11999 (Figure 2). For environments which had sufficiently abundant PF20597 and PF11999 sequences, this model had 93.3%, 85.3% and 71.3 % accuracy in frozen sediment, rock and subsurface environments respectively. This comparable accuracy despite lack of sequence homology between the two protein families demonstrates the power of the alignment-free, structurally-aware transfer learning approach used in our main model. PF20597 was not included in our final model to allow comparison to alignment-based approaches such as the GA, as well as to allow interpretation of results in a structure-function context, for which detailed information on PF20597 is not available.

It is important to note that the positional logistic regression accuracy described in the text may not be directly comparable to our ANN as it utilises an alignment based approach which implicitly violates test/training independence. Importantly, our ANN uses alignment-free encoding - specifically, embeddings from the ESM model - which does not require the sequences to be of equal length or to share sequence similarity. This enables the model to be applied broadly to variable and potentially divergent sequences. In contrast, any method that uses positional identity directly as input, such as the LR-PI model or our genetic algorithm

approach, inherently requires sequences to be the same length. This can be achieved through trimming, padding, or alignment (sequence or structure), which has several downsides: Loss of biological signal: Padding or trimming may remove or obscure meaningful features, especially in variable regions that are important for function or environmental adaptation. Bias introduced by alignment: Misalignments or poor-quality regions can introduce noise or bias into the input representation, especially where there is a large amount of diversity in the dataset. Reduced applicability: Positional models may perform well on tightly aligned training datasets but are not readily extendable to new sequences that differ in length or structure, limiting utility.

Another key consideration when comparing these approaches is the computational resources required. Generating ESM-2 embeddings is fast once the pretrained model is loaded, and memory usage can be scaled to available resources by adjusting the batch size and embedding dimensions. This makes the method scalable and suitable for processing large sets of variable-length, diverse sequences.

In contrast, generating multiple sequence alignments (MSAs) for HMMs or positional encoding approaches is often significantly slower and more memory-intensive, especially for large datasets with long or highly divergent sequences.

Once suitable sequence representations have been obtained from ESM-2, alignments, or one-hot encodings, the difference in feasibility lies in hardware requirements. Building and training an artificial neural network (ANN) typically requires a GPU, which may not be readily available on a standard desktop computer. However, alternatives such as Google Colab offer free access to GPUs, making small-scale model development feasible. Because our method uses a relatively shallow ANN (two layers), it remains computationally lightweight and can be

run on such platforms. That said, deeper networks would increase resource demands accordingly.

Meanwhile, generating MSAs for large, divergent datasets is also challenging on local machines and quickly becomes infeasible without access to high-performance computing resources.

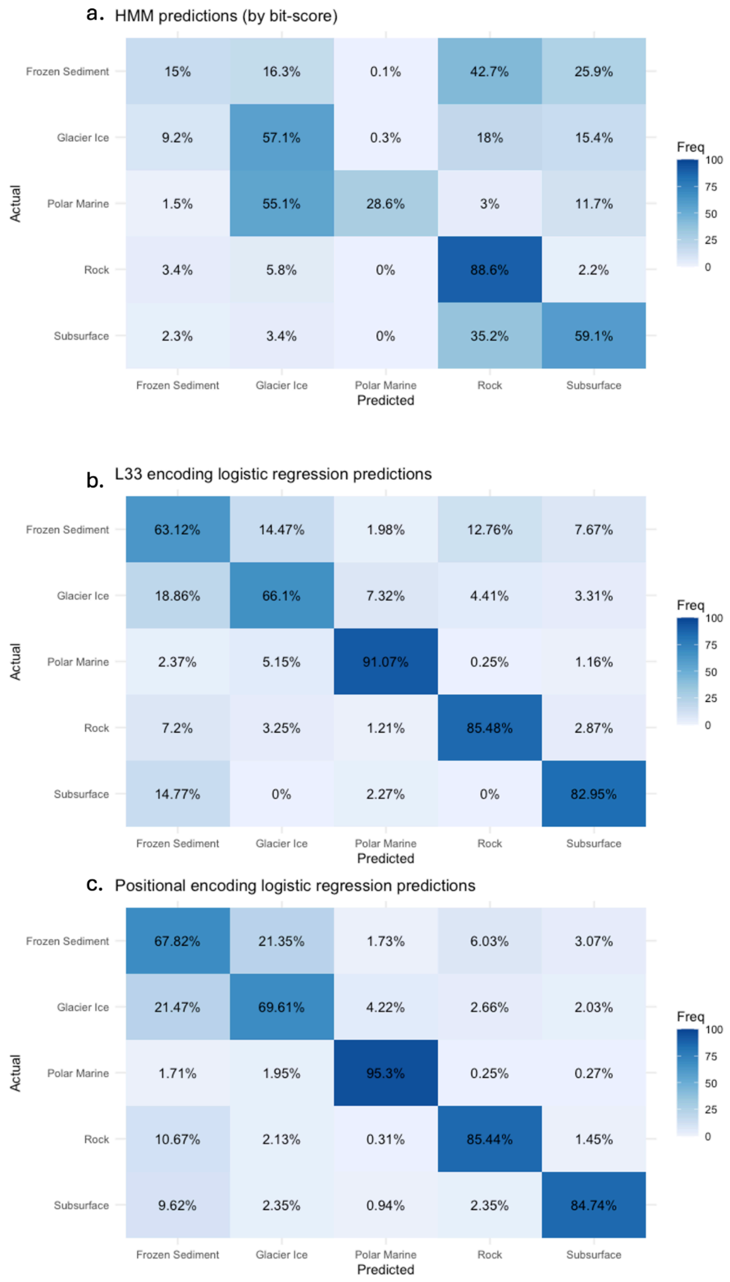

Figure 1: Confusion matrices for 3 different sequence classification approaches. a. Hidden Markov Model approach whereby 80% of sequences for each environment were aligned (within environments) and used to build hmm profiles, and the remaining 20% were aligned against the whole profile. The profile hit with the highest bit score was taken as the predicted environment. b. Multinomial logistic regression with encodings from layer 33/34 of ESM (L33). c. Multinomial logistic regression with one-hot encoded positional identity of the multiple sequence alignment.

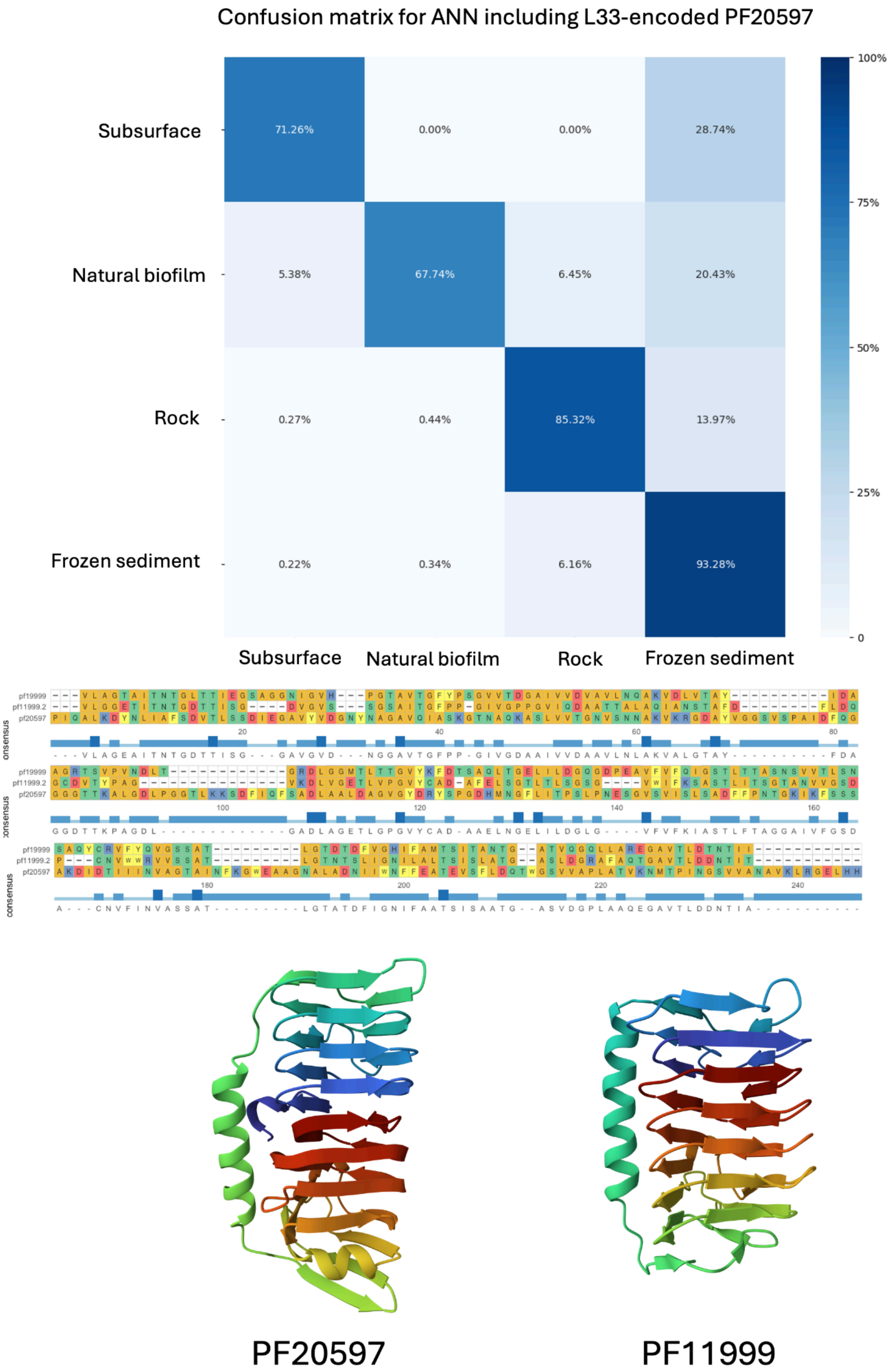

Figure 2: Results for an ANN which included both PF20597 and PF11999. (a). Confusion matrix shows comparable accuracy to the PF11999-only model for frozen sediment, rock and subsurface. (b) Representative PF11999 and PF20597 DUF3494 sequences align poorly to one another compared to PF11999 sequences to themselves. Shared residues between the 2 PF11999 sequences are in light blue, while those shared between the 2 PF11999 sequences

and the PF20597 sequence are shown in dark blue. (c) PF20597 and PF11999 DUF3494s are structurally similar but not identical.
