# Supplementary material for: Environmental adaptations in metagenomes revealed by deep learning
Source: BMC Biol. 2025 Aug 11;23:252. doi: 10.1186/s12915-025-02361-1 (PMC12337378; doi:10.1186/s12915-025-02361-1)
Supplement: Supplementary file 4 — Additional file 4: Supplementary Figures. [file 12915_2025_2361_MOESM4_ESM.pdf]

Additional File 4: Supplementary Figures

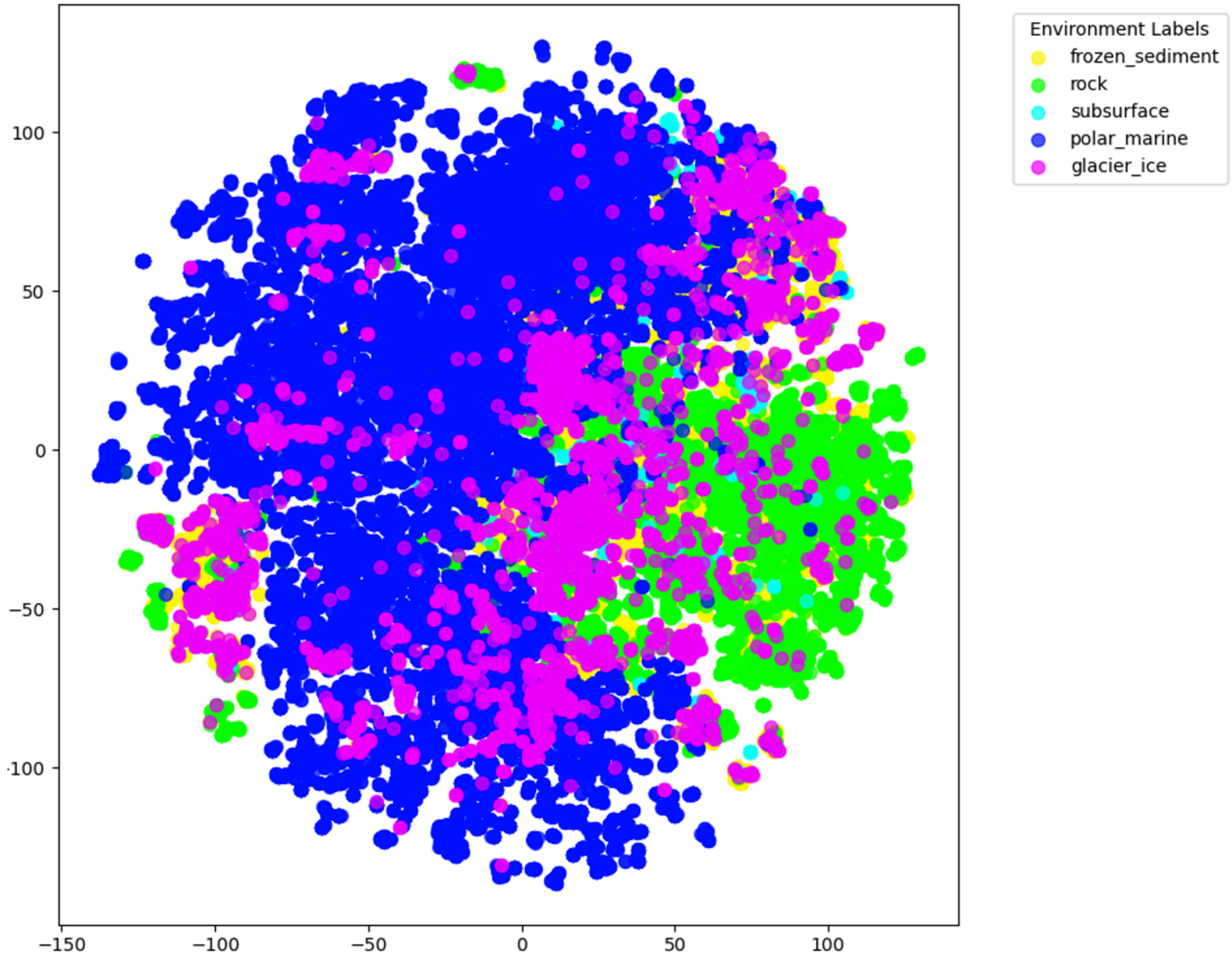

Figure 1: t-SNE plot of ESM-2 encoded DUF3494 sequences does not show obvious clusters.

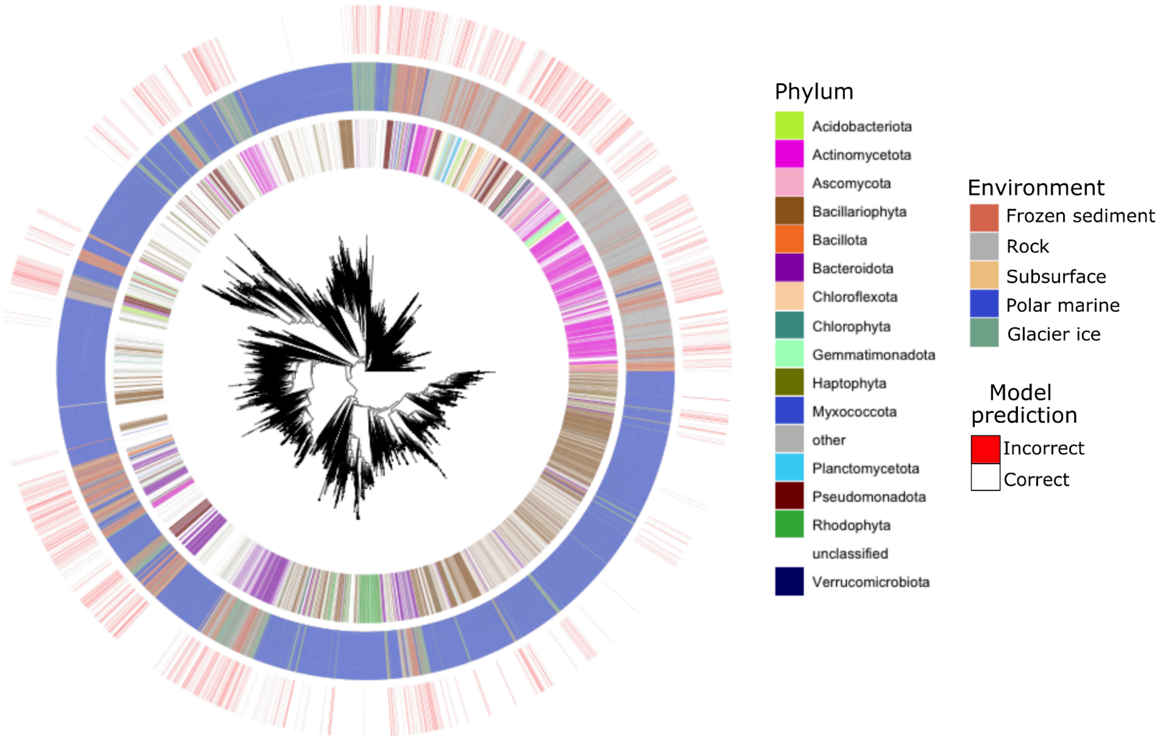

Figure 2: Phylogenetic tree of DUF3494 sequences annotated with taxonomy, environment, and model correctness. Inner ring is phylum-level classification, with unclassified sequences not coloured. Most abundant phyla are coloured per-phylum, while less abundant phyla are coloured in grey. Middle ring is environment: red = frozen sediment, grey = rock, yellow = subsurface, blue = polar marine, green = glacier ice. Outer ring is model correctness - red where the model incorrectly predicted the environment.
