# Supplementary material for: Environmental adaptations in metagenomes revealed by deep learning
Source: BMC Biol. 2025 Aug 11;23:252. doi: 10.1186/s12915-025-02361-1 (PMC12337378; doi:10.1186/s12915-025-02361-1)
Supplement: Supplementary file 5 — Additional file 5: Results. [file 12915_2025_2361_MOESM5_ESM.pdf]

Additional File 5: Results

2.2 Important residues:

In addition to diversity differences between environments, we also examined specific positions and amino acids which varied between environments. PF11999 DUF3494s are often ice-binding proteins, and to this end are expected to have ice-binding sites. These sites have been found on the b-face and c-face of DUF3494 proteins [1, 2]. While these are not necessarily the same in every protein, they form flat planes which contain linearly aligned side chains down the vertical axis of the protein. These columns of aligned side-chains might occur multiple times on the ice-binding face, and the distance between coils of the solenoid (rows) nearly matches the distance between water molecules on basal and prism planes of hexagonal ice [3]. The first column has been shown to include hydrophobic amino acids with small side chains such as alanine (A), and glycine (G) while a second column contains hydrophilic amino acids with small side chains containing hydroxyl groups such as threonine (T) and serine (S) which might contribute to hydrogen bonding. Finally, the c-face has been less specifically implicated in ice-binding. An exploration of 50 randomly sampled protein structures across environments showed frequent columns of aligned hydrophilic side chains (T and S). To test whether this was generalisable across the whole protein, we identified which aligned residues corresponded to these vertical “columns” of residues along the faces of the protein. We then performed a chi-squared test on the abundance of all amino acids in this column, compared to the abundance of these amino acids in the rest of the protein (expected values were the expected averages for seven residues). Due to the large sample size, the p-value was significant for all positions tested despite large differences in the □^{2} statistic. We therefore compared the effect size (Cramer’s V) for each column too (Table 3). As we were interested in positions where a small number of AAs (either T/S or A/G) were dominant, we also calculated the Shannon diversity index (H) of each position. We then selected the three columns with the highest

proportion of their most abundant AA (Figure 5e) and performed per-environment calculations on these columns.

2.3 Column 2 (b-face)

The first column of interest (b-face, col_2) mapped to the alignment positions 6, 26, 177, 158, 140, 113 and 87 (Figure X). At position 6, Shannon diversity (sdiv) varied from 1.46 (rock) to 1.85 (glacier ice), and the number of amino acids found varied from 13 (subsurface) to 20 (frozen sediment, polar marine, glacier ice). At position 26, sdiv varied from 1.25 (rock) to 2.14 (subsurface), with between 12 (subsurface) and 21 (frozen sediment, glacier ice) AAs. At position 177, sdiv varied from 1.05 (rock) to 1.48 (subsurface), ranging from 10 AAs (subsurface) to 21 (frozen sediment). At position 158, sdiv varied from 1.07 (rock) to 1.79 (frozen sediment and glacier ice), with between 14 (subsurface) and 21 (frozen sediment) AAs. At position 140, sdiv ranged from 0.79 (rock) to 1.81 (subsurface), with between 15 (subsurface) and 22 (frozen sediment) AAs. At position 113, sdiv varied from 1.03 (rock) to 1.70 (frozen sediment) with between 10 (subsurface) and 21 (glacier ice) AAs. Finally, at position 87 sdiv varied from 1.62 (polar marine) to 1.91 (frozen sediment) with between 12 (subsurface) and 20 (frozen sediment, glacier ice) AAs.

In column 2 (b-face), average Shannon diversity was lowest closer to the middle of the sheet (positions 177, 158, 149, 113) (Figure 5a). While rock sequences had the lowest diversity at each of these individual positions, the amino acids at these positions were inconsistent down the column of residues, with hydrophobic As at 26 and 177, and hydrophilic Ts and Ss at other positions. This inconsistency was mirrored by frozen sediment and subsurface environments, in alignment with the misclassification pattern from the ANN (above). Conversely, in more “pure ice” environments such as glacier ice and polar marine (sea ice and seawater), this column was

consistently dominated by hydrophobic As frequently preceded by Gs, consistent with it being an ice-binding site in these environments.

2.4: Column 2

The second column of interest (b-face, col_4) mapped to the aligned positions 28, 8, 179, 160, 142, 115 and 89, which are also vertically aligned along the centre of the b-face of the protein. At position 28, sdiv varied from 0.95 (rock) to 1.73 (polar_marine) with between 12 (subsurface) and 20 (frozen sediment) AAs. At position 8, sdiv varied from 0.77 (rock) to 1.70 (glacier ice), with between seven (subsurface) and 20 (glacier ice and polar marine) AAs. At position 179, sdiv varied from 0.96 (rock) to 1.85 (polar marine) with between 10 (subsurface) and 21 (polar marine) AAs. At position 160, sdiv varied from 1.33 (rock) to 1.94 (glacier ice) with between 14 (subsurface) and 22 (glacier ice) AAs. At position 142, sdiv varied from 0.69 (rock) to 1.87 (polar marine) with between 12 (subsurface) and 21 (polar marine) AAs. At position 115, sdiv varied from 1.53 (rock) to 2.03 (polar marine) with between 12 (subsurface) and 20 (polar marine, frozen sediment) AAs. At position 89, sdiv varied from 1.78 (polar marine) to 2.43 (frozen sediment) with between 16 (subsurface) and 21 (polar marine and frozen sediment) AAs.

Like the first putative IBS, rock sequences consistently had the lowest Shannon diversity. Rock sequences down this column were consistently dominated by hydrophobic residues, specifically threonine, and at position 160, threonine or serine. Once again, subsurface and frozen sediment sequences had similar patterns, with subsurface consistently having the smallest number of AAs. Meanwhile, for polar marine and glacier ice sequences, although threonine/serine were still the single most frequent AAs, these were a much smaller proportion of the total residues, with a variety of other amino acids also being found. This second column of aligned residues is thought to be involved with hydrogen bonding. Taken with the results from the first column of

residues, this could imply that these hydrophobic interactions could be more important in the function of DUF3494 proteins in non-pure ice environments.

2.5: Column 3

The third column of interest (a-face, col_0) mapped to the aligned positions 35, 17, 169, 151, 133, 106 and 80. At position 35, sdiv varied from 0.13 (subsurface) to 1.11 (polar marine) with between five (subsurface) and 21 (polar marine) AAs. At position 17, sdiv varied from 0.25 (subsurface) to 0.75 (glacier ice), with between four (subsurface) and 20 (polar marine) AAs. At position 169, sdiv varied from 0.14 to 0.46 (glacier ice) with between three (subsurface) and 14 (polar marine) AAs. At position 151, sdiv varied from 0.07 (subsurface) to 0.15 (glacier ice) with between three (subsurface) and nine (frozen sediment) AAs. At position 133, sdiv varied between 0.47 (rock) and 0.93 (subsurface) with between six (subsurface and glacier ice) and 11 (rock) positions. At position 106 sdiv varied from 0.37 (rock) to 0.87 (glacier ice) with between six (subsurface) and 12 (frozen sediment) AAs. At position 80 sdiv varied from 0.17 (polar marine) and 0.36 (glacier ice) with between six (subsurface) and 13 (rock and frozen sediment) AAs.

Shannon diversity was overall lowest in this column. It was not consistently lowest in any given environment. This is likely because the composition of this position was consistent within and between environments. In all environments, the first four columns were dominated by G, the 5th column contained nonpolar side chain I or V, the 6th column was dominated by aromatic hydrophobic AAs F and W, and the 7th column was dominated by G. The proportions of F:W differed slightly between environments, with F dominating in rock environments.

2.5: Insertion found in rock sequences

We identified a potential insertion/deletion which was more prevalent in rock environments compared to other environments. At aligned positions 66-68 (between the bottom of the alpha helix and the bottom sheet of the b-face), sequences in polar marine, glacier ice, subsurface and frozen sediment environments had >34 % of gaps (polar marine: 34.4% ± 14.1%; glacier ice: 41.6% ± 7.4%; subsurface: 45.4±0.1%; frozen sediment: 40.6% ± 2.3%) while in rock environments, just 12.5%±2.6% of values at these positions was gaps. Instead, in rock environments, the most abundant AAs were T or A at position 66 (17.9%;15.3%), L or V at position 67 (39.6%; 24.8%) and T or P at position 68 (33.0%; 13.1%). Rock sequences are one of the most easily distinguishable sequences by the ANN and this is mirrored by their lower local and overall diversity, as well as this potential insertion.

3. Genetic algorithm

a-face:

On the a-face, distinguishing features of sequences from frozen sediment environments often included positions 81, 83 (bottom sheet of a-face), and 106 (2nd to bottom sheet of a-face). Amino acids which frequently (>1 rule included) appeared in rules for this environment were leucine (L) at position 81 (24.5% of sequences in this environment; 7.9% of sequences in other environments), a cysteine at position 83 (18.6%; 7.2%), the absence of phenylalanine (F) (46.5%; 22.0%) or presence of tryptophan (W) (39.9%; 18.7%) at position 106, and an F at position 108 (87.8%; 65.7%).

Polar marine sequence rules often included positions 108, 133 and 134 (3rd from bottom loop of a-face). AAs which frequently appeared in rules for this environment were isoleucine (I) at position 105 (21.7% of sequences in this environment; 8.1% of sequences in other environments), and I at position 134 (9.9%;1.3%).

Rock sequence rules often included positions 106, 132 and 134. AAs which frequently appeared in rules for this environment were F at position 106 (90.7% of sequences in this environment; 71.6% of sequences in other environments), E at position 132 (0.7%; 0.2%), and the absence of L (99.9%; 95.8%) and the absence of W (99.8%; 99.9%) at position 134.

Glacier ice sequence rules often included positions 105, 106, 107 and 133. AAs which frequently appeared in rules for this environment were T at position 105 (7.4% of sequences in this environment; 4.4% of sequences in other environments), presence of W (37.0%; 20.1%) and absence of F (42.7%;23.7%) at position 106, presence of valine (V) at position 133 (76.3%; 66.2%).

Subsurface sequence rules often included positions 80 and 107. The AA which was the most frequent across rules for this environment was an H at position 80 (2.4%; 1.4%).

Rules pertaining to residues found on the a-face (helix-facing) of the protein often included very hydrophobic (L, I, F, W, V) AAs with bulky side chains. Across this face, the strongest signal was for the frozen sediment environment, and the weakest was for subsurface environments.

b-face:

On the b-face, frozen sediment rules often included positions 71, 72 and 145. The AA which was the most frequent across rules for this environment was the absence of A at position 145 (86.1% of sequences in this environment; 48.7% of sequences in other environments).

Polar marine sequence rules often included positions 71, 72 and 145. The AAs which most frequently appeared in rules for this environment were A at position 145 (63.8% of sequences in

this environment; 19.3% of sequences in other environments), V at position 71 (11.0%;1.9%) and the absence of L at position 72 (83.3%; 32.8%).

Rock sequence rules often included positions 71 and 72. The AAs which most frequently appeared in rules for this environment were D at position 71 (63.3%; 13.8%) and L at position 72 (84.1%; 28.0%).

Glacier ice sequence rules often included positions 71, 73 and 144. The AA which was the most frequent across rules for this environment was D at position 73 (1.9%; 1.3%).

Subsurface sequence rules often included positions 26 and 71. The AA which was the most frequent across rules for this environment was the absence of V at position 27 (93.4%; 81.6%).

Rules for positions on the b-face (ice-binding) of the protein often included the absence (and presence) of specific hydrophobic AAs (A, L, V). This is interesting especially when bulky AAs such as L have been shown to decrease ice-binding activity of the DUF3494s on this surface. Conversely, small hydrophobic residues like A are an important component of the IBS. Across this face, the strongest signals were for polar marine and rock, and the weakest was for subsurface.

c-face:

On the c-face, frozen sediment rules most often included positions 77 and 97. The AA which was the most frequent across rules for this environment was the absence of D at position 97 (49.8%; 25.8%).

Polar marine sequence rules most often included positions 94, 95 and 77. The AA which was the most frequent across rules for this environment was M at position 77 (0.9%; 0.3%).

Rock sequence rules rarely included positions on the c-face with rules appearing at positions 7, 78, 79, 96 and 97 but no position having a frequency of >1.

Glacier ice sequence rules most often included positions 78 and 97. The AA which was the most frequent across rules for this environment was N at position 78 (3.4%; 3.0%) .

Subsurface sequence rules most often included positions 78, 79 and 95. The AAs which most frequently appeared in rules for this environment were the absence of K (100%; 97.9%), and the absence of R (100%; 99.3%), at position 78, the absence of A at position 79 (90.8%; 86.3%), the absence of Y (97.7%; 95.4%) and absence of V (99.1; 95.6) at position 95.

Rules for positions the c-face were generally quite rare and had weak signals. Subsurface rules had the most components, however these were all absence rules whose predictive power is inherently weaker (less specific) than presence rules. The functional role of this face is less clear and these results support the hypothesis that it may play an indirect role, such as protein stability.

between faces:

On conserved regions between faces, frozen sediment rules most often included position 153. The AA which was the most frequent across rules for this environment was the absence of L at position 153 (91.7% of sequences in this environment; 72.1% of sequences in other environments).

Polar marine sequences rules only included position 152, with the rule being the presence of I at this position (11.6%; 5.9%).

Rock sequence rules included positions 124 and 173, of which position 124 was more frequent. All AAs had the same frequency in rules in this environment.

Glacier ice sequence rules most often included positions 126, 171 and 173. The AAs which most frequently appeared in rules for this environment were G at position 171 (8.8% of sequences in this environment; 0.9% of sequences in other environments), and the absence of A at position 173 (65.4%; 53.7%).

Subsurface rules most often included positions 125, 152 and 173. The AA which was the most frequent across rules for this environment was A at position 173 (81.7%; 44.9%)

The most frequent positions appearing in frozen sediment rules were positions 106 (a-face), 71, 72, and 145 (b-face). Across these rules, the most common elements were C at position 83, the absence of F at position 106, and the absence of A at position 145

The most frequent positions appearing in polar marine rules were positions 71, 72 and 145 (b-face). Across these rules, the most common elements were A at position 145, V at position 71 and the absence of L at position 72.

The most frequent positions appearing in rock rules were positions 71 and 72 (b-face). Across these rules, the most common elements were D at position 71, L at position 72, and F at position 106.

The most frequent positions appearing in glacier ice rules were positions 105, 106 (a-face), and 73 (b-face). Across these rules, the most common elements were the absence of F at 106, the presence of W at 106, the presence of G at 171 and the absence of A at 173 (between faces).

The most frequent positions appearing in subsurface rules were positions 78, 95 (c-face), 26, 71 (b-face) and 107 (a-face). Across these rules, the most common element was the absence of K at position 78.

Position 71 was a common component of rules in all environments except glacier ice. For rock and polar marine rules, single amino acids dominated these positions. In the context of aligned hydrophobic/hydrophilic residues along ice-binding face of the protein (as discussed above), it is notable that in rock environments rules this position was most often dominated by a hydrophobic AA (valine) while in polar marine rules this was a neutral/hydrophilic AA (aspartate).

Rules for positions between faces varied, with the presence of specific hydrophobic AAs (I, G and A) being the most consistent trend. The functional implications of this may follow on from the same effect seen on the a-face.

Additional File 6: Supplementary discussion

Further discussion of environmental adaptation of DUF3494

Type and higher concentrations of solute can also enhance ice-recrystallisation activity [4], so it is possible that in these high-solute environments, there is less pressure to optimise protein structure. Alternatively, it is possible that this increased diversity is reflecting more relaxed selection or more functional divergence in these proteins, in favour of different ecological roles. For example, in addition to binding ice, certain non-DUF3494 ice-binding proteins have been shown to inhibit the growth of other crystals such as α-d-mannopyranoside [5]. Alternatively, rather than binding ice, it is possible that these proteins bind water, as this is an especially scarce resource in permafrost environments [6, 7]. Indeed, proteins in the PF20597 family have the same discontinuous B-solenoid/braced alpha helix shape as DUF3494, and they have been implicated in biofilm formation by diatoms [8] and human pathogens [9, 10], a process that involves binding of water [11](Chang & Halverson, 2003). Our ANN struggled to distinguish between glacier ice and frozen sediment more than between other environments. This could potentially imply less phylogenetic constraints and more functional adaptation in these environments.

References:

1. Mangiagalli M, Bar-Dolev M, Tedesco P, Natalello A, Kaleda A, Brocca S, et al. Cryo-protective effect of an ice-binding protein derived from Antarctic bacteria. FEBS J. 2017;284:163–77. https://doi.org/10.1111/febs.13965.

2. Wang C, Pakhomova S, Newcomer ME, Christner BC, Luo B-H. Structural basis of antifreeze activity of a bacterial multi-domain antifreeze protein. PLOS ONE. 2017;12:e0187169. https://doi.org/10.1371/journal.pone.0187169.

3. Vance TDR, Bayer-Giraldi M, Davies PL, Mangiagalli M. Ice-binding proteins and the 'domain of unknown function' 3494 family. FEBS J. 2019;286:855–73. https://doi.org/10.1111/febs.14764.

4. Yu SO, Brown A, Middleton AJ, Tomczak MM, Walker VK, Davies PL. Ice restructuring inhibition activities in antifreeze proteins with distinct differences in thermal hysteresis. Cryobiology. 2010;61:327–34. https://doi.org/10.1016/j.cryobiol.2010.10.158.

5. Wang S, Wen X, DeVries AL, Bagdagulyan Y, Morita A, Golen JA, et al. Molecular Recognition of Methyl α-d-Mannopyranoside by Antifreeze (Glyco)Proteins. J Am Chem Soc. 2014;136:8973–81. https://doi.org/10.1021/ja502837t.

6. Hinsa-Leasure SM, Koid C, Tiedje JM, Schultzhaus JN. Biofilm Formation by Psychrobacter arcticus and the Role of a Large Adhesin in Attachment to Surfaces. Appl Environ Microbiol. 2013;79:3967–73. https://doi.org/10.1128/AEM.00867-13.

7. Steven B, Léveillé R, Pollard WH, Whyte LG. Microbial ecology and biodiversity in permafrost. Extremophiles. 2006;10:259–67. https://doi.org/10.1007/s00792-006-0506-3.

8. Suchanova JZ, Bilcke G, Romanowska B, Fatlawi A, Pippel M, Skeffington A, et al. Diatom adhesive trail proteins acquired by horizontal gene transfer from bacteria serve as primers for marine biofilm formation. 2023;:2023.03.06.531300. https://doi.org/10.1101/2023.03.06.531300.

9. Ma Z, Sun Y, Liu Y, Jiao J, Li N, Zuo Y, et al. STM1863, a Member of the DUFs Protein Family, Is Involved in Environmental Adaptation, Biofilm Formation, and Virulence in Salmonella Typhimurium. Foodborne Pathog Dis. 2024. https://doi.org/10.1089/fpd.2023.0139.

10. Xu Y, Liang X, Chen Y, Koehler TM, Höök M. Identification and Biochemical Characterization of Two Novel Collagen Binding MSCRAMMs of Bacillus anthracis*. J Biol Chem. 2004;279:51760–8. https://doi.org/10.1074/jbc.M406417200.

11. Chang W-S, Halverson LJ. Reduced Water Availability Influences the Dynamics, Development, and Ultrastructural Properties of Pseudomonas putida Biofilms. J Bacteriol. 2003;185:6199–204. https://doi.org/10.1128/jb.185.20.6199-6204.2003.
