# Supplementary material for: Environmental adaptations in metagenomes revealed by deep learning
Source: BMC Biol. 2025 Aug 11;23:252. doi: 10.1186/s12915-025-02361-1 (PMC12337378; doi:10.1186/s12915-025-02361-1)
Supplement: Supplementary file 6 — Additional file 6: Supplementary discussion. [file 12915_2025_2361_MOESM6_ESM.pdf]

Additional File 6: Supplementary discussion

Further discussion of environmental adaptation of DUF3494

Type and higher concentrations of solute can also enhance ice-recrystallisation activity [1], so it is possible that in these high-solute environments, there is less pressure to optimise protein structure. Alternatively, it is possible that this increased diversity is reflecting more relaxed selection or more functional divergence in these proteins, in favour of different ecological roles. For example, in addition to binding ice, certain non-DUF3494 ice-binding proteins have been shown to inhibit the growth of other crystals such as α-d-mannopyranoside [2]. Alternatively, rather than binding ice, it is possible that these proteins bind water, as this is an especially scarce resource in permafrost environments [3, 4]. Indeed, proteins in the PF20597 family have the same discontinuous B-solenoid/braced alpha helix shape as DUF3494, and they have been implicated in biofilm formation by diatoms [5] and human pathogens [6, 7], a process that involves binding of water [8](Chang & Halverson, 2003). Our ANN struggled to distinguish between glacier ice and frozen sediment more than between other environments. This could potentially imply less phylogenetic constraints and more functional adaptation in these environments.

References:

1. Yu SO, Brown A, Middleton AJ, Tomczak MM, Walker VK, Davies PL. Ice restructuring inhibition activities in antifreeze proteins with distinct differences in thermal hysteresis. Cryobiology. 2010;61:327–34. https://doi.org/10.1016/j.cryobiol.2010.10.158.

2. Wang S, Wen X, DeVries AL, Bagdagulyan Y, Morita A, Golen JA, et al. Molecular Recognition of Methyl α-d-Mannopyranoside by Antifreeze (Glyco)Proteins. J Am Chem Soc. 2014;136:8973–81. https://doi.org/10.1021/ja502837t.

3. Hinsa-Leasure SM, Koid C, Tiedje JM, Schultzhaus JN. Biofilm Formation by Psychrobacter arcticus and the Role of a Large Adhesin in Attachment to Surfaces. Appl Environ Microbiol. 2013;79:3967–73. https://doi.org/10.1128/AEM.00867-13.

4. Steven B, Léveillé R, Pollard WH, Whyte LG. Microbial ecology and biodiversity in permafrost. Extremophiles. 2006;10:259–67. https://doi.org/10.1007/s00792-006-0506-3.

5. Suchanova JZ, Bilcke G, Romanowska B, Fatlawi A, Pippel M, Skeffington A, et al. Diatom adhesive trail proteins acquired by horizontal gene transfer from bacteria serve as primers for marine biofilm formation. 2023;:2023.03.06.531300. https://doi.org/10.1101/2023.03.06.531300.

6. Ma Z, Sun Y, Liu Y, Jiao J, Li N, Zuo Y, et al. STM1863, a Member of the DUFs Protein Family, Is Involved in Environmental Adaptation, Biofilm Formation, and Virulence in Salmonella Typhimurium. Foodborne Pathog Dis. 2024. https://doi.org/10.1089/fpd.2023.0139.

7. Xu Y, Liang X, Chen Y, Koehler TM, Höök M. Identification and Biochemical Characterization of Two Novel Collagen Binding MSCRAMMs of Bacillus anthracis*. J Biol Chem. 2004;279:51760–8. https://doi.org/10.1074/jbc.M406417200.

8. Chang W-S, Halverson LJ. Reduced Water Availability Influences the Dynamics, Development, and Ultrastructural Properties of Pseudomonas putida Biofilms. J Bacteriol. 2003;185:6199–204. https://doi.org/10.1128/jb.185.20.6199-6204.2003.
